# Surface morphology and distribution of oropharyngeal taste papillae in sharks and rays (Elasmobranchii, Chondrichthyes): Implications for gustatory sensitivity

**DOI:** 10.1111/joa.14278

**Published:** 2025-07-18

**Authors:** Carla J. L. Atkinson, Shaun P. Collin

**Affiliations:** ^1^ School of Biomedical Sciences The University of Queensland Brisbane Queensland Australia; ^2^ The School of Agriculture, Biomedicine and Environment La Trobe University Bundoora Victoria Australia; ^3^ Oceans Graduate School and Oceans Institute The University of Western Australia Crawley Western Australia Australia; ^4^ Max Planck Queensland Centre (MPQC) for the Materials Science of Extracellular Matrices Queensland University of Technology Kelvin Grove Queensland Australia

**Keywords:** chemoreception, gustation, microvilli, oral papilla, taste bud

## Abstract

Gustation or taste in elasmobranch fishes (sharks, skates, and rays) is an important sensory modality that dictates the palatability and ultimately the final decision regarding the ingestion of food. However, the surface morphology, size, abundance, and distribution of taste papillae in this group of apex predators has received little attention. This comparative study uses scanning electron microscopy, histology, and quantitative topographic analyses to assess the surface ultrastructure and density of taste papillae within the oropharyngeal cavity of six batoid species from three families and five selachian species from three families, all from a range of habitats and with a variety of diets. Within the batoids, mean taste papilla diameter ranges from 56 to 220 μm (with 0.7–1.6% of the papilla surface covered with sensory microvilli), while papilla diameter ranges from 152 to 360 μm in selachians (with 0.4–1.0% of the papilla surface covered with sensory microvilli). Both batoids and selachians possess two distinct size classes of papillae within the oropharyngeal cavity, where up to five small papillae (56–62 μm in diameter) often surround a large papilla (159–192 μm in diameter). There are significant differences in the total number of taste papillae within the oropharyngeal cavity in both superorders of elasmobranchs with a range of 2,119–20,317 in batoids (papillae occupying up to 3.1% of the oropharyngeal cavity with 0.05% of the cavity occupied by sensory microvilli) and a range of 1,354–11,890 in selachians (papillae occupying up to 1.7% of the oropharyngeal cavity with 0.02% of the cavity occupied by sensory microvilli) with taste papillae generally concentrated in areas used for food mastication. In batoids, papillae concentrate on ridges within the oropharyngeal cavities and in some species also on the oral valves (47–175 cm^−1^ in the dorsal cavity, 33–160 cm^−1^ in the ventral cavity). In selachians, the highest concentrations of taste papillae are on the oral valves and anterior regions of the oral cavity (4–215 cm^−1^ in the dorsal cavity; 5–159 cm^−1^ in the ventral cavity), which permits taste assessment during biting and manipulation of potential food items. This study is the first to investigate the abundance and distribution of taste papillae in the oropharyngeal cavity of a range of species of elasmobranchs, thereby improving our understanding of the importance of gustation, implications for oral food manipulation, and interpretations of both gustatory resolution and sensitivity.

## Graphical abstract

This study investigates the morphology, abundance, and distribution of taste papillae in the oropharyngeal cavity of a range of elasmobranchs using SEM, histology, and topographic analyses. The interspecific importance of gustation is reflected in quantitative differences in the size, density, and distribution of taste papillae, which have implications for oral food manipulation and prey palatability and provide proxies for gustatory resolution and sensitivity.

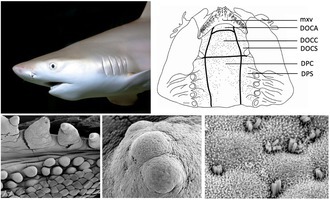

## 1 INTRODUCTION

Taste buds are the gustatory sense organs in vertebrates that orally process and evaluate the palatability of food through direct contact, eventually leading to a decision to either ingest or reject it. The process is fundamental to life as consumed food provides important energy necessary for processes, such as growth, locomotion, neural activity, and the maintenance of a healthy immune system (Collin & Atkinson, 2025; Finger, 2007). In all vertebrates, taste buds comprise modified epithelial cells generally forming pear‐shaped structures located on the top of raised papillae typically located within the oropharynx (Reutter, 1982; Reutter, 1992). Taste buds are composed of receptor cells, support cells, and basal cells and are innervated by the facial (VII), glossopharyngeal (IX), and vagal (X) cranial nerves (Finger, 1997; Reutter et al., 2000).

In fishes (both bony and cartilaginous), taste buds are known to occur within papillae of the oral and pharyngeal epithelia of the mouth, basihyal (‘tongue’), oral valves and gill arches, and their morphology appears comparable in these two major groups (Reutter et al., 1974). Taste buds have been also identified on the external surface of teleost fishes including over the head, fins, and barbels (Atema, 1971; Bardach & Atema, 1971; Gomahr et al., 1992; Kapoor et al., 1975) and in some teleost species, taste buds form rows oriented antero‐posteriorly within the palate (Hara et al., 1994; Marui et al., 1983). To date, no gustatory nerves have been found to innervate the trunk of elasmobranchs (Sheldon, 1909), suggesting that they lack external taste buds.

Four morphological types of taste buds have been characterized in teleost fishes, each differentiated by the distance they protrude above the surrounding epithelium and size. Type I taste buds protrude the furthest and are surrounded by a shallow depression at their base. Type II taste buds are similar to Type I but do not have the depression, and Type III taste buds lie within a pore on an otherwise flat region of cornified, desquamating epithelium (Reutter et al., 1974). Miniature Type IV taste buds are reported in a number of cardinal fish species (*Apogon* sp.) (Fishelson et al., 2004). Taste buds in teleosts are ~100 μm high and 40 μm wide (Reutter & Witt, 2004) and may possess an epithelial pore between 5 and 8 μm in diameter (Reutter et al., 1974). However, taste bud morphology varies considerably across species, and little is known regarding their function (Kapoor et al., 1975).

Elasmobranchs (sharks, skates, and rays) represent one of the earliest groups of extant jawed vertebrates (gnathostomes) and can therefore provide important information about the evolution of the gustatory system. However, the morphology of taste buds has only been examined in a small number of elasmobranch species, and no studies exist on their distribution within the oropharynx. Two and three types of taste buds have been identified in the oropharynx (‘tongue’, palate and gill bars) of the lesser‐spotted dogfish, *Scyliorhinus canicula*, by Todaro (1872) and Bateson (1890), respectively, while basal cells and sensory cells bearing apical microvilli have also been confirmed as taste buds of the southern stingray, *Trygon pastinaca*, and the thornback ray, *Raja clavata*, by Pevsner (1976). Ultrastructural investigations of taste buds (including supporting and basal cells) have also been made in *Scyliorhinus canicula* (Reutter, 1994; Whitear & Moate, 1994b), *Squalus acanthias* (Cook & Neal, 1921) and *Heterodontus* sp. (Daniel, 1934). More recently, a comparison of the surface ultrastructure of oral papillae in four species of lamnid and sphyrnid sharks reveals that the shape, size, and arrangement of oral papillae (and denticles) may be related to the ecology and phylogeny of each species (Rangel et al., 2019). In the porbeagle shark, *Lamna nasus*, the density of oral papillae was found to be approximately 100 per cm^2^ in the ventral region of the oropharyngeal cavity, with a clear inverse relationship between the density of oral papillae and oral denticles (Poscai et al., 2022). A similar relationship was found for six species of requiem sharks (Carcharhinidae) with interspecific variations in the size (57.6–182.7 μm in diameter) and density (100–400 cm^−2^ and 125–260 cm^−2^ in the dorsal and ventral regions of the oropharyngeal cavity, respectively) of oral papillae (Poscai et al., 2021).

Ontogenetic differences of taste papillae morphology in the brown‐banded bamboo shark, *Chiloscyllium punctatum*, have been studied, where the oral valve papillae have been shown to be comparable to Type I taste buds of teleost fishes, whereas taste buds located in other regions of the oropharyngeal cavity are comparable to Type II taste buds. Both types of papillae in *C. punctatum* show immunofluorescence for a number of markers of taste buds, including β‐Catenin and Sox2 (Atkinson et al., 2016). Three other species of elasmobranchs (the red stingray, *Hemitygon akajei*, the sepia stingray, *Urolophus aurantiacus* and the spiny dogfish, *Squalus suckleyi*) all show immunoreactivity to serotonin in the basal and elongate cells of their taste buds, which may modulate responses of taste receptor cells (Ikenaga et al., 2023). Although not an elasmobranch but a member of the cartilaginous group of fishes (Chondrichthyes), the holocephalan, *Chimaera monstrosa*, possesses G‐protein‐alpha‐subunit‐inhibitory‐like (Gαi‐like) immunoreactivity within the elongate cells of all taste buds over the ‘tongue’ (Ferrando et al., 2012). These taste buds are different from those located within the palatal organ of a diversity of holocephalan species, which is thought to play a part in food sorting and processing (Ferrando et al., 2016; Finucci et al., 2018).

With respect to the abundance and distribution of taste buds in fishes, teleosts possess some of the highest total numbers of oropharyngeal taste buds in vertebrates with between 6,600 (in minnows, Kiyohara et al., 1980) to 24,600 (in blennies and gobies, Fishelson et al., 2004). In comparison, the number of taste buds is low in other groups of vertebrates that is 1,400–2,000 in the axolotl (Northcutt et al., 2000), 316 in chickens (Ganchrow & Ganchrow, 1985), 1,265 in rats (Miller, 1977), 723 in hamsters, and 195 in humans (Cheng & Robinson, 1991). Peak densities of taste buds located within the oral cavity of teleosts also vary, that is, from 300 to 500 cm^−2^ in the catfish, *Ictalurus natalis* (Atema, 1971) and 1,500 cm^−2^ in the minnow, *Pseudorasbora parva* (Kiyohara et al., 1980) to 17,000 cm^−2^ in the tench, *Tinca tinca* (Fishelson et al., 2004) and over 30,000 cm^−2^ in some cyprinids (Gomahr et al., 1992). Analyses of taste bud distribution provide information about the functional and neuroecological adaptations of gustation and identify species‐specific differences in feeding mechanisms, oral food manipulation, and any correlations with diet and habitat (Berkhoudt, 1992; Gon et al., 2007). The abundance of taste buds in teleosts has also been associated with the ability to effectively locate a food source (Bardach et al., 1967; Boudriot & Reutter, 2001), although it is unknown whether differences in the total number or distribution of taste buds relate to gustatory sensitivity (Caprio, 1988).

There are only a few studies on the abundance and distribution of taste buds in elasmobranchs (Poscai et al., 2021, 2022). In the developing oropharyngeal cavity of the bamboo shark, *Chiloscyllium punctatus*, the density of Type I taste buds is highest within the oral valves, with a density of 2,125–3,483 cm^−2^ in embryos compared with 89–111 cm^−2^ in mature adults (Atkinson et al., 2016), while the overall number of taste buds (1,900) remains constant throughout development. Atkinson and Collin (2012) state that the number of protective oral denticles (scales) in elasmobranchs appears to be inversely proportional to the number of taste papillae and that the distribution of denticles, which are frequently found to touch or overlap in their distribution, may often account for a low density of taste papillae. The highest density (180 cm^−2^) of papillae was also observed in the maxillary and mandibular valve regions of different stages of ontogenetic growth within the oral cavity of the blue shark, *Prionace glauca* (Rangel et al., 2017).

In addition to aggregations of taste receptors within taste bud papillae, solitary chemosensory cells (SCCs), which are innervated by either the trigeminal nerve or a spinal nerve, are also found in most gnathostomatous vertebrates (Collin & Atkinson, 2025; Kotrschal, 1991, 1996). These SCCs are secondary epidermal sensory cells, which resemble taste receptors (Whitear, 1965) but do not form part of a distinct organ or bud, where they are embedded between unspecialized epidermal cells. SCCs are found in cyclostomes, teleost fishes, and elasmobranchs (Collin & Atkinson, 2025; Whitear & Moate, 1994a, 1994b). In most species, SCCs are scattered over the body surface, including the oral cavity.

In the present study, we investigate the surface morphology, size, and proportionate coverage within the oropharyngeal cavity of taste papillae in six batoid (ray) species from three families and five selachian (shark) species from three families using histology and scanning electron microscopy (SEM). The abundance and topographic distribution (mean density) of taste papillae within the oropharyngeal cavities are also described, along with the proportion of the oropharyngeal cavity occupied by gustatory tissue, to provide an anatomical proxy of sensitivity and improve our understanding of the feeding process, oral prey manipulation, and the ingestion of food in species with a range of diets and from a diversity of habitats, representing the two major groups of elasmobranchs.

## 2 MATERIALS AND METHODS

### 2.1 Collection and preservation of animals

All specimens (see Table 1), except *Trygonorrhina fasciata*, *Taeniura lymma*, and *Trygonorrhina dumerilii*, were caught in Australia by seine netting or hand netting within the regions of Moreton Bay in Southeast Queensland or Heron Island on the Great Barrier Reef, Australia. *T. fasciata* and *T. lymma* were purchased from commercial suppliers (Cairns Marine, Cairns and Pet City, Brisbane, Australia respectively), while *T. dumerilii* was purchased from a local collector, who obtained one individual (180 mm TL) from Port Phillip Bay, Victoria, Australia. All animals were anaesthetized with MS 222 (tricaine methane sulfonate salt 1:250, Sigma) and immediately decapitated anterior to the pectoral fins by severing just posterior to the heart to include all the oral epithelium (oropharynx) but minimal oesophageal tissue. Heads were immersion‐fixed in Karnovsky's fixative (2% paraformaldehyde, 2.5% glutaraldehyde, 2.2% sodium cacodylate, pH 7.4). All procedures followed the ethical guidelines of The University of Queensland Animal Ethics Committee (AEC Number: ANAT/978/08/ARC [NF]) and the La Trobe University Animal Ethics Committee (AEC Number 22019).

**TABLE 1:** List of all species of elasmobranchs used in the study, including their sizes in mm (disc width (DW), disc length (DL) and total length (TL)), their habitat, diet and depth range. Information from Fishbase (fishbase.org) and Last and Stevens (2009).

Family (common name), *Species* (common name)	Dimensions (mm)	Habitat	Diet	Depth range (m)
Trygonorrhinidae (Guitarfishes)				
*Aptychotrema rostrata* (eastern shovelnose ray)	*n* = 1, TL 750	Benthic on shallow parts of estuaries and beaches	Shrimps and crabs, molluscs, teleost fishes, cephalopods	0–220
*Trygonorrhina fasciata (*eastern fiddler ray)	*n* = 1, DW 180, DL 189, TL 416	Benthic in shallow water	Shrimps and crabs, teleost fishes, cephalopods	0–100
*Trygonorrhina dumerilii* (southern fiddler ray)	*n* = 1, DW 85, DL 95, TL 190	Benthic on soft bottoms and seagrasses	Crustaceans, worms, molluscs and small fishes	5–205
Dasyatidae (Stingrays)				
*Hemitrygon fluviorum* (estuary stingray)	*n* = 1, DW 383, DL 353, TL 918	Benthopelagic on sand flats, mangroves and estuaries	Crustaceans (crabs), shellfish, polychaete worms	0–40
*Neotrygon kuhlii* (blue‐spotted mask ray)	*n* = 1, DW 363, DL 290, TL unknown	Benthic on shallow sand flats near rocky and coral reefs, shallow lagoons at high tide	Crabs, shrimps, polychaete worms, small teleost fishes	0–170
*Taeniura lymma* (blue‐spotted fantail ray)	*n* = 1, DW 169, DL 184, TL 418	Benthic on shallow coral reefs, shallow lagoons at high tide	Molluscs, worms, shrimps, crabs	0–20
Gymnuridae (Butterfly rays)				
*Gymnura australis* (Australian butterfly ray)	*n* = 1, DW 399, DL 210, TL 296	Benthic on open sandy or muddy and silty estuaries and intertidal zones	Mostly teleost fishes, crustaceans and cephalopods	150–250
Orectolobidae (Carpet sharks)				
*Orectolobus maculatus* (spotted wobbegong shark)	*n* = 2, TL 674, 835 *n* = 2, TL 1,160, 1,172	Benthic on coral and rocky reefs, feeding on sand flats at night, nocturnal	Crabs, lobsters, reef fishes, octopuses	0–248
*Orectolobus ornatus* (ornate wobbegong shark)	*n* = 2, TL 507, 623	Benthic on coral and rocky reefs, nocturnal	Benthic invertebrates, teleost fishes	0–100
Hemiscylliidae (Bamboo sharks)				
*Hemiscyllium ocellatum* (epaulette shark)	*n* = 3, TL 585, 624, 655	Benthic on shallow water reefs, tidal pools	Worms, crabs, shrimps, small teleost fishes, molluscs	0–50
Carcharhinidae (Requiem sharks)				
*Carcharhinus melanopterus* (black‐tip reef shark)	*n* = 2, TL 933, 1,030	Pelagic over shallow water reefs, sand flats, mangroves	Teleost fishes, cephalopods, crustaceans, molluscs	0–75
*Negaprion acutidens* (sickle‐fin lemon shark)	*n* = 2, TL 1,209, 1,380	Pelagic over shallow reefs, sandy lagoons, turbid mangrove swamps	Teleost fishes, small sharks and stingrays	0–92

*Note*: *Trygonorrhina dumerilii* was used only for illustrating the major external and internal features and for orientation within the oropharynx.

**FIGURE 1:**
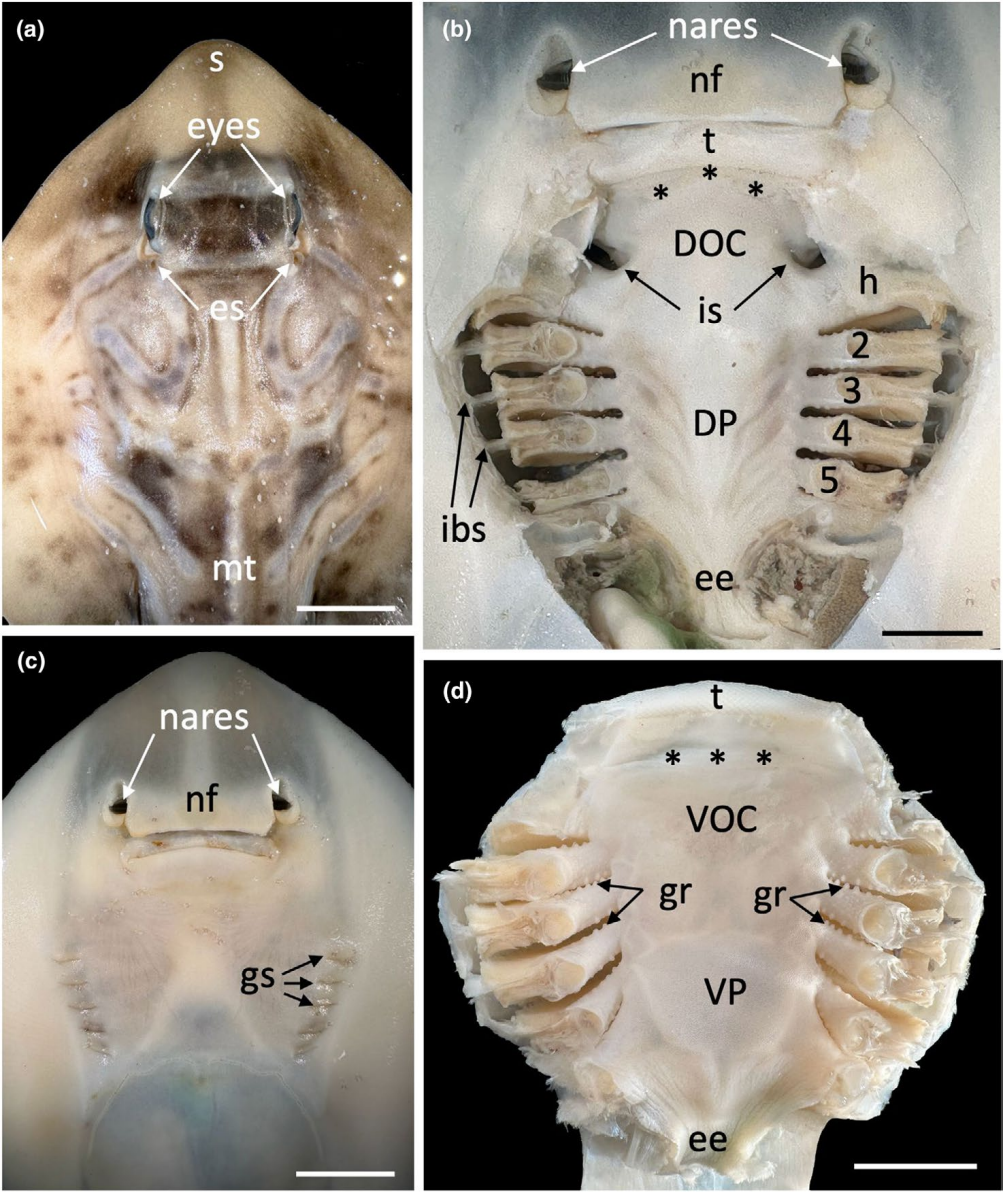
Gross morphology of the external and internal anatomy of the southern fiddler ray, *Trygonorrhina dumerilii* showing the typical approach to the dissections for anatomical investigations and assessment of the distribution of taste papillae. (a) Dorsal view of the head of *T. dumerilii* showing the position of the eyes, snout (s), external spiracles (es) and mid‐line tubercles (md). (b) Inner surface of the maxilla showing the regional location of various structures. DOC, dorsal oral cavity; DP, dorsal pharynx; ee, eosophageal entrance; h, hyoid or 1st gill arch; 2, 2nd gill arch; 3, 3rd gill arch; 4, 4th gill arch; 5, 5th gill arch; ibs, interbranchial septa; is, internal spiracles; nf, nasal fold; t, teeth. Asterisks depict the location of the maxillary oral valve (abbreviated as mxv in distribution maps). (c) Ventral view of *T. dumerilii* showing the position of the nares, nasal fold (nf) and the external gill slits (gs). (d) Inner surface of the mandible showing the regional location of various structures. ee, eosophageal entrance; gr, gill rakers; t, teeth; VOC, ventral oral cavity; VP, ventral pharynx. Asterisks depict the location of the mandibular oral valve (abbreviated as mdv in distribution maps). Scale bars: 2 cm (a); 1 cm (b); 2 cm (c); 1 cm (d).

Six species of (rays) batoids and six species of sharks (selachians) were used in the study, with between one and four individuals sampled for each species. The batoids included two species from the family Trygonorrhinidae: the eastern shovelnose ray *Aptychotrema rostrata* (Shaw, 1794) (*n* = 1, total length (TL) 750 mm) and the eastern fiddler ray *Trygonorrhina fasciata* (Müller & Henle, 1841) (*n* = 2, disc width (DW) 180–189 mm, disc length (DL) 189–196 mm, TL 416 mm), three species from the family Dasyatidae: the estuary stingray *Hemitrygon fluviorum* (Ogilby, 1908) (*n* = 1, DW 383 mm, DL 353 mm, TL 918 mm), the blue‐spotted mask ray *Neotrygon kuhlii* (Müller & Henle, 1841) (*n* = 1, DW 363 mm, DL 290 mm, TL unknown), and the blue‐spotted fantail ray *Taeniura lymma* (Forsskål, 1755) (*n* = 1, DW 169 mm, DL 184 mm, TL 418 mm), and one species from the family Gymnuridae: the Australian butterfly ray *Gymnura australis* (Ramsay & Ogilby, 1886) (*n* = 2, DW 399–475 mm, DL 210–252 mm, TL 296–330 mm). The southern fiddler ray *Trygonorrhina dumerilii* was only used to illustrate the external and internal (oropharyngeal cavity) features and introduce the nomenclature (Figure 1). The selachians included two species from the family Orectolobidae: the spotted wobbegong *Orectolobus maculatus* (Bonnaterre, 1778) (*n* = 4, TL 674–1,172 mm) and the ornate wobbegong *Orectolobus ornatus* (De Vis, 1883) (*n* = 3, TL 507–629 mm), one species from the family Hemiscylliidae: the epaulette shark *Hemiscyllium ocellatum* (Bonnaterre, 1788) (*n* = 4, TL 585–655 mm), and two species from the family Carcharhinidae: the black‐tip reef shark *Carcharhinus melanopterus* (Quoy & Gaimard, 1824) (*n* = 3, TL 933–1,030 mm) and the sickle‐fin lemon shark *Negaprion acutidens* (Rüppell, 1837) (*n* = 2, TL 1,209–1,380 mm). Information regarding habitat and diet is provided by Fishbase (fishbase.org) and Last and Stevens (2009), and, along with a taxonomic and size data, is presented in Table 1. Observations of locomotory behaviour and feeding were carried out on individuals kept in large aquaria (up to 4 m in diameter) prior to euthanasia or while snorkelling.

### 2.2 Histological processing of gustatory tissue

To assess the morphology of selected taste papillae in transverse section at the level of both the light microscope and the scanning electron microscope (SEM), regions of the epithelium were dissected from all regions of the oropharyngeal cavity and prepared for histological processing. The dorsal (maxillary) and ventral (mandibulary) regions of the oropharyngeal cavity were divided into seven topographic regions on the basis of mouth morphometrics, in addition to the maxillary and mandibulary oral valves (as performed previously for assessing the structure and topography of oropharyngeal denticles in elasmobranchs by Atkinson & Collin, 2012). Therefore, the nomenclature for the identification of each region closely follows Atkinson and Collin (2012); mxv, maxillary oral valve; DOCA, dorsal oral cavity anterior; DOCC, dorsal oral cavity centre; DOCS, dorsal oral cavity side (×2); DPC, dorsal pharynx centre; DPS, dorsal pharynx side (×2); mdv, mandibulary oral valve; VOCA, ventral oral cavity anterior; VOCC, ventral oral cavity centre; VOCS, ventral oral cavity side (×2); VPC, ventral pharynx centre; VPS, ventral pharynx side (×2). Two pieces of oropharyngeal epithelium roughly 1 × 1 cm were also dissected from the centre of each region. Tissue samples for observations using light microscopy were embedded in paraffin wax. Processing involved removing the tissue from the fixative and placing it in 0.1 M cacodylate buffer for 15 min. The tissue was then dehydrated in a graded series of ethanols (70%, 90%, 100%, 100%) for 45 min each, placed into two different containers of xylene for 45 min each, immersed into wax at 60°C for 45 min, then into new wax at 60°C in a vacuum at 4.08 atmospheres for 45 min (Thermo Scientific EC 350 Paraffin Embedding Centre, Thermo Fisher Scientific Inc.) and then finally embedded in paraffin blocks. Serial sections (4 μm thick) were cut using a Hyrax M25 Rotary Microtome (Carl Zeiss MicroImaging GmbH, Germany) and mounted onto subbed glass slides and stained with haematoxylin and eosin or Toluidine blue.

Tissue samples destined for analysis using SEM were processed using a microwave tissue processor (Biowave® PELCO International CA USA). Samples were rinsed in 0.1 M sodium cacodylate buffer in a vacuum at 80 Watts (W) for 40 s and postfixed in 1% osmium tetroxide in 0.1 M cacodylate buffer in a vacuum at 80 W for 2 min on and 2 min off, repeated three times. Samples were then dehydrated in a graded series of ethanols (50%, 70%, 90%, 100%, 100%) at 250 W for 40 s each and infiltrated with hexamethyldisilazane (HMDS) (1:1 with 100% ethanol, then twice in 100% HMDS) at 250 W for 40 s each, then left to dry overnight. Samples were platinum‐coated (8 nm) in an Eiko IB‐5 Ion Coater (Eiko Engineering Company, Japan) and examined using a JEOL JSM 6300F Scanning Electron Microscope (JEOL LTD. Tokyo, Japan) at 5 kV.

### 2.3 Quantitative and topographic analyses of taste papillae

The dimensions of taste papillae and microvilli were recorded from both light and scanning electron micrographs and analysed using Image Processing and Analysis software in Java (ImageJ) (Table 2). Surface areas of the papillae were measured from micrographs of papillae taken perpendicular to the epithelial surface within the oropharyngeal cavity. Taste papillae were characterized as a circular region atop slightly elevated cells, which were clearly differentiated from the surrounding flat squamous epithelium and contained apical microvilli. Please note surface areas were not corrected for three dimensions and represented the two dimensional area of the taste papilla coverage as a proportion of the total surface area of the oropharynx when viewed from a position directly overhead. Mean papillae diameter was calculated ± standard errors (SE) and ANCOVA and Tukey's pairwise comparison tests were used to determine any significant differences. Wherever possible, taste papillae were characterized according to morphological criteria described for teleosts, by Reutter et al. (1974) and Fishelson et al. (2004), that is, Type I protrude the furthest from the epithelium and are surrounded by a shallow depression at their base; Type II are similar to Type I but do not have the depression; Type III lie within a pore on an otherwise flat region of cornified, desquamating epithelium and Type IV are relatively much smaller.

**TABLE 2:** Summary of the quantitative data on mean papilla diameter within the oropharynx ± SE and number sampled, the mean proportion (%) of each taste papillae occupied by microvilli, the total number of papillae, the mean proportion (%) of the oropharynx occupied by taste papillae, the mean proportion (%) of the oropharynx occupied by gustatory sensory tissue, and the area of the oropharynx occupied by gustatory sensory tissue.

Superorder, *species*	Mean papilla diameter ± SE (μm)	Mean % of papilla occupied by microvilli	Total number of papillae	Mean % area covered by papillae	Mean % gustatory area	Gustatory area (μm^2^)
Batoids						
*A. rostrata*	146 ± 9 (*n* = 11)	1.5	8,630	2.429	0.036	2,167,474
*T. fasciata*	104 ± 0.4 (*n* = 8)	1.6	4,015	1.305	0.021	545,781
*H. fluviorum*	56 ± 1 (*n* = 88)	1.6	9,343	3.127	0.050	3,855,035
	159 ± 4 (*n* = 34)		10,974			
*N. kuhlii*	62 ± 4 (*n* = 17)	1.5	4,553	2.808	0.042	2,500,016
	192 ± 7 (*n* = 11)		5,281			
*T. lymma*	143 ± 6 (*n* = 12)	0.7	2,950	2.879	0.020	331,695
*G. australis*	220 ± 6 (*n* = 13)	0.7	2,119	2.323	0.016	563,924
Selachians						
*O. maculatus*	254 ± 7 (*n* = 30)	0.4	1,916	1.400	0.006	775,873
	359 ± 13 (*n* = 31)					
*O. ornatus*	256 ± 21 (*n* = 14)	0.5	1,993	1.312	0.007	512,984
*H. ocellatum*	175 ± 6 (*n* = 130)	1.6	1,354	1.151	0.018	521,148
*C. melanopterus*	203 ± 4 (*n* = 61)	0.7	9,235	1.764	0.012	2,092,537
*N. acutidens*	152 ± 5 (*n* = 44)	1.0	11,890	0.900	0.009	2,157,820

*Note*: The two values for mean papillae diameter in *H. fluviorum, N. kuhlii*, and *O. maculatus* represent the ability to clearly and consistently differentiate small and large papillae. The total number of papillae in *H. fluviorum* and *N. kuhlii* is 20,317 and 9,834, respectively.

**FIGURE 2:**
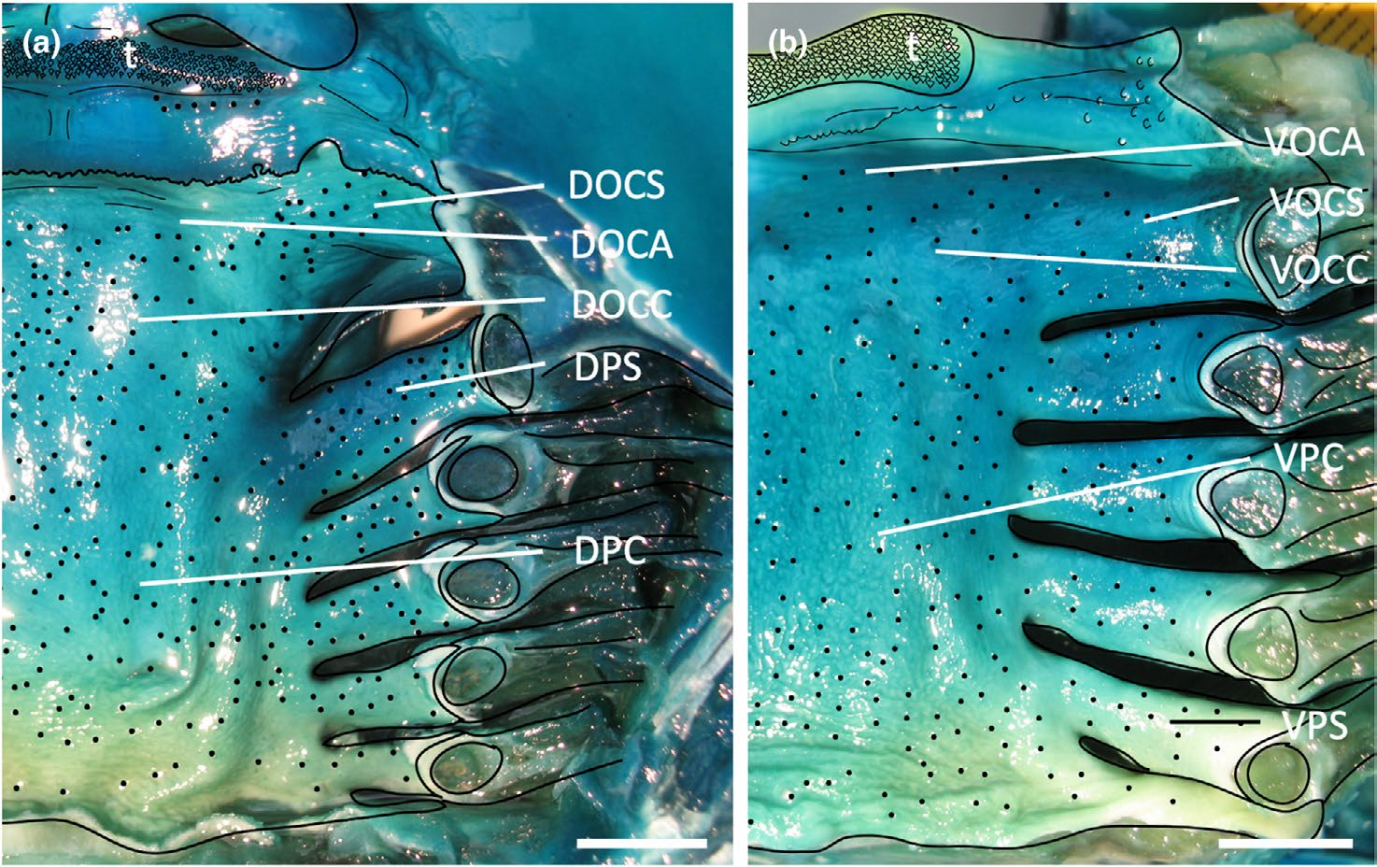
Photographs of the dissected oropharyngeal regions of the maxilla (a) and mandible (b) in one individual of *Gymnura australis* following the application of methylene blue stain. Dots have been overlayed to represent the position of prominently stained taste papillae observed at the level of the light microscope. Lines depict the positions of the gill arches and the boundary with the tooth array (t). See Figure 6 for other abbreviations. See Figure 15 for the boundaries of each region. Maxillary and mandibulary oral valves are not shown. Scale bars 1.0 cm (a, b).

For estimating the topographic distribution of taste papillae within the oropharynx, heads were cut at the juncture of the hyomandibular, ceratohyal, lower jaw, and palatoquadrate cartilages (Wilga et al., 2007) and separated into dorsal (maxillary) and ventral (mandibular) parts and placed in methylene blue overnight. Upper and lower jaws (including pharynx) were placed separately in either methylene blue or Toluidine blue solution (0.01% in 0.1 M phosphate buffer at 25°C) for between 15 and 30 min or until all oropharyngeal epithelia were stained (Figure 2). Methylene blue has been found to preferentially stain taste buds in both teleosts and mammals, with the receptor region of the taste bud having the highest affinity due to the accumulation of mucus (Müller & Reutter, 1995). Re‐staining was sometimes necessary to enhance contrast. Methylene blue and Toluidine blue stain the taste papillae and apical nerve endings more intensely than the surrounding epithelium, an approach that has been independently confirmed using molecular characterization of taste papillae with immunofluorescence in bamboo sharks (*Chiloscyllium punctatus*) (Atkinson et al., 2016). Dorsal and ventral oropharyngeal linings in each region were then photographed with a Sony Cybershot DWC‐W200 camera, and papillae were counted within various topographic regions on digital images using Image Processing and Analysis software in Java (ImageJ 1.38x) with a cell_counter.jar plugin before the mean densities and standard errors (SE) for each species were calculated to the nearest whole number. SEM was also used to assess the abundance and density of taste papillae at higher magnifications, which uncovered the presence of smaller taste papillae not observed using the stain‐enhanced light micrographs.

Topographic maps presented for each species show the outlines of the dorsal (maxillary) and ventral (mandibular) regions traced from the digital light micrographs. Each dot represents a prominent taste papilla as observed following the methylene blue/Toluidine blue enhancement method (Figure 2). However, the densities listed for each zone: mxv and mdv (where present), DOCA, DOCC, DOCS, DPC, DPS, VOCA, VOCC, VOCS, VPC, and VPS, in Table 3 include the smaller taste papillae (assessed using SEM). Therefore, although useful with respect to identifying the relative densities of large taste papillae within the oropharynx in each species, the density of taste papillae in these distribution plots is graphically underrepresented. The distribution maps shown in Figures 6, 23 and 24 do not reveal the locations of prominent taste papillae due to the large size of these individual species and the inability to map the location of each prominent taste papilla on each outline and still differentiate the individual dots at the scale shown. Therefore, refer to Table 3 for the mean densities of all types of taste papillae in localized zones within the dorsal and ventral regions of the oral cavity and pharynx.

**TABLE 3:** Mean density of taste papillae cm^−2^ ± SE for the maxillary oral valve (mxv), the mandibular oral valve (mdv) and the different regions of the oral cavity (anterior, central, side) and pharynx (central side) in both dorsal and ventral areas in each species examined.

Superorder, *species*	Dorsal						Ventral					
	Oral cavity				Pharynx		Oral cavity				Pharynx	
	mxv	Anterior	Central	Side	Central	Side	mdv	Anterior	Central	Side	Central	Side
Batoids												
*A. rostrata*	—	160 ± 1.2	130 ± 1.3	100 ± 1.7	145 ± 0.9	135 ± 0.7	—	160 ± 1.3	110 ± 1.1	110 ± 1.3	155 ± 1.0	95 ± 1.0
*T. fasciata*		114 ± 9	143 ± 25	130 ± 7	159 ± 26	159 ± 29	—	100 ± 16	107 ± 21	157 ± 5	138 ± 9	151 ± 8
*H. fluviorum*	—	77 ± 5	79 ± 18	87 ± 10	64 ± 7	119 ± 15	—	80 ± 2	33 ± 5	75 ± 17	53 ± 4	76 ± 12
*N. kuhlii*	—	81 ± 1	77 ± 7	60 ± 2	47 ± 14	96 ± 8	—	91 ± 6	61 ± 3	91 ± 4	72 ± 6	67 ± 7
*T. lymma*	—	160 ± 5	143 ± 6	175 ± 17	120 ± 2	165 ± 22	—	55 ± 4	85 ± 3	73 ± 1	88 ± 5	80 ± 6
*G. australis*	—	63 ± 0.5	58 ± 0.5	65 ± 0.6	50 ± 0.5	60 ± 0.4	—	65 ± 0.4	73 ± 0.4	65 ± 0.5	68 ± 0.3	68 ± 0.4
Selachians												
*O. maculatus*	31 ± 7	14 ± 5	4 ± 2	21 ± 8	5 ± 2	18 ± 8	33 ± 12	11 ± 3	7 ± 3	15 ± 4	5 ± 2	18 ± 4
*O. ornatus*	84 ± 6	39 ± 7	20 ± 3	50 ± 7	13 ± 1	40 ± 7	61 ± 13	25 ± 14	18 ± 7	25 ± 8	13 ± 2	38 ± 4
*H. ocellatum*	110 ± 3	103 ± 21	49 ± 6	83 ± 15	61 ± 10	52 ± 9	56 ± 3	63 ± 9	26 ± 4	45 ± 5	30 ± 3	49 ± 3
*C. melanopterus*	215 ± 19	58 ± 14	20 ± 7	29 ± 4	10 ± 1	55 ± 12	159 ± 4	38 ± 5	24 ± 6	39 ± 5	33 ± 6	58 ± 13
*N. acutidens*	83 ± 3	116 ± 28	28 ± 8	75 ± 2	37 ± 1	54 ± 7	90 ± 14	40 ± 15	29 ± 6	37 ± 2	29 ± 3	45 ± 10

Mean surface areas for papillae were calculated (using πr^2^) and collectively represented as a percentage of the oropharyngeal region that is occupied by sensory receptors, as has previously been conducted for the bamboo shark *Chiloscyllium punctatum* (Atkinson et al., 2016). The total sensory area was calculated to the nearest whole number using the equation: ((PA*TP)/100)*S, where PA is the papilla area (πr^2^), TP is the total number of papillae, and S is the percentage of the papilla that is covered in sensory microvilli. No attempt was made to calculate the surface area of the microvilli emanating from solitary chemosensory cells.

## 3 RESULTS

The epithelium lining the oral and pharyngeal cavities of all species of sharks and rays examined is comprised of a mosaic‐like pavement of pentagonally‐ and hexagonally stratified, squamous epithelial cells (~10 μm in diameter), each adorned with dense aggregations of microvilli and some microplicae over their surface. Taste papillae are found throughout the oropharyngeal regions of all species analysed, and unless stated otherwise, their diameter did not differ significantly throughout the oropharynx at the 5% significance level. Scanning electron microscopy of the surface ultrastructure and light microscopy of transverse sections of individual taste papillae reveal that taste papillae are circular in shape, possess a differentiated region at their apical tip (within either a slight depression or a protrusion) from which tufts of microvilli project. These differentiated regions are either centrally located or dispersed over the apical surface of the papilla. Based on morphological criteria, the taste buds do vary in diameter (large and small diameter papillae are differentiated in *H. fluviorum*, *N. kuhlii* and *O. maculatus*) and height, with taste papillae located on the maxillary and mandibular oral valves projecting further into the oropharynx. Some attempt was made to assign taste papillae into types found in teleosts according to Reutter et al. (1974), that is, Type I taste buds protrude the furthest and are surrounded by a shallow depression at their base; Type II taste buds are similar to Type I but do not have the basal depression; and Type III taste buds lie within a pore on an otherwise flat region of cornified, desquamating epithelium. However, this was not always possible due to the variation in the range of sizes of each species and the inability to accurately assess the height of the taste papillae above the epithelium. No external taste buds were identified, but a systematic histological examination of the dermis was not carried out for any species.

**FIGURE 3:**
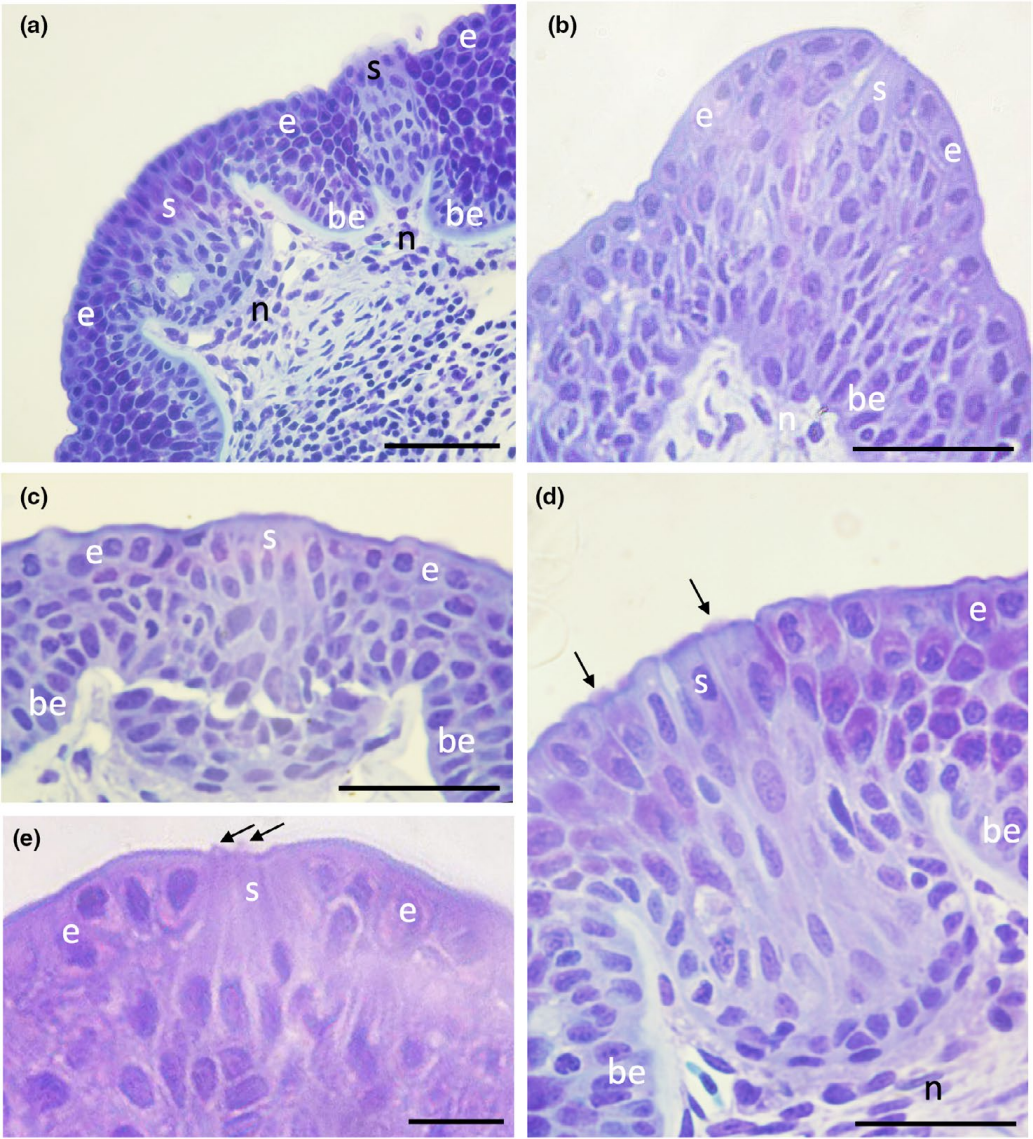
Light micrographs of taste papillae in transverse section in different species of elasmobranchs. (a) Apical zone of a taste papilla in *Hemitrygon fluviorum* showing multiple regions of differentiated pear‐shaped structures within the squamous epithelium (e). Note numerous sensory taste receptors (s) send projections to the surface. (b) Dome‐shaped taste papilla in *Hemiscyllium ocellatum*. (c) Sensory taste receptor projections (s) within a flat‐topped taste papilla in *Taeniura lymma*. (d) The apical zone of a taste papilla in *H. fluviorum* showing multiple sensory projections (s) from tufts of microvilli (arrows) protruding through the surface epithelium (e). (e) Taste papilla in *H. ocellatum* showing tufts of microvilli at the surface from large, underlying sensory cells (s). be, basal epithelium; n, nerve fibre layer. Scale bars: 50 μm (a); 100 μm (b); 50 μm (c); 20 μm (d); 20 μm (e).

In transverse section, taste papillae appeared similar across species but with a diversity of size (diameter), height above the epithelial surface, the shape and convexity of the apical region, the number of taste buds per taste papillae, the number of receptor cells within each taste bud, the presence of apical microvilli, and the arrangement of sensory receptor cells, the basal epithelium, and the nerve fibre layer (Figure 3). However, this level of variation has also been noted in previous studies on the characterization of taste buds using histological (Reutter, 1994) and molecular techniques (Atkinson et al., 2016).

The following subsections arranged into families provide a species‐specific description of both the morphology and topographic distribution of taste papillae within the oropharynx. Where appropriate, some brief descriptions are also included for teeth and denticles. Figure 1 provides a guide to the major external and internal landmarks of the oropharynx in the southern fiddler ray, *Trygonorrhina dumerilii*, but which can also be used for all species in the following descriptions of the morphology and distribution of taste papillae.

### 3.1 Guitarfishes (Trygonorrhinidae)

#### 3.1.1 Morphology of taste papillae

**FIGURE 4:**
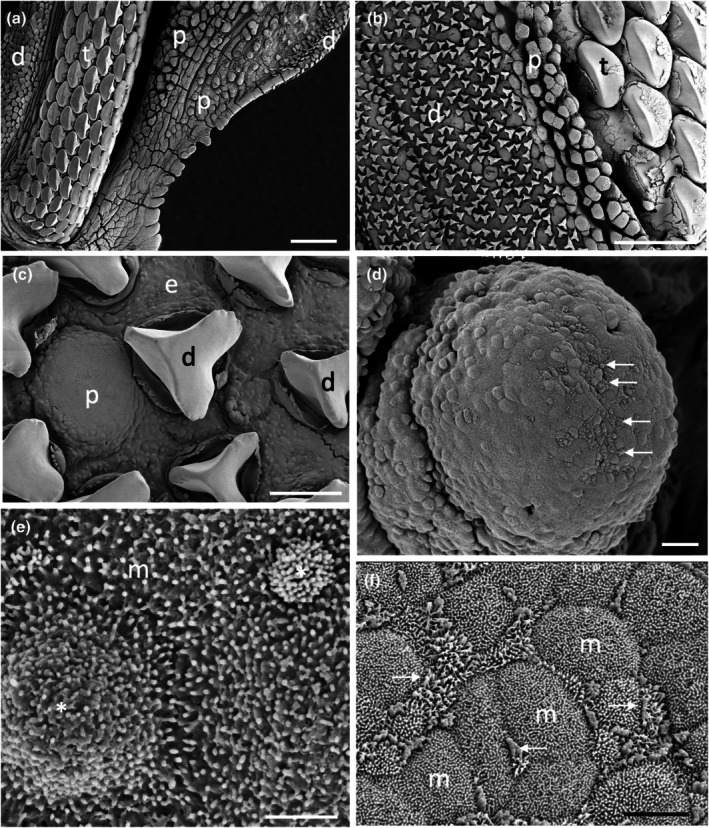
Taste papillae in the Eastern shovelnose ray, *Aptychotrema rostrata* viewed using scanning electron microscopy. (a) Taste papillae (p) over the maxillary (a) and mandibular (b) oral valves. Note the dense aggregations of denticles (d) and teeth (t). (c) A taste papilla (p) protruding from the flat squamous epithelium (e) of the oropharyngeal region located amongst triangular denticles. (d) Raised taste papillae showing several tufts of microvilli (arrows) protruding from the surface of the epithelium. (e) Bulbous projections (asterisks) on the flat squamous epithelium covered with microvilli (m). (f) Aggregations of long microvilli (arrows) protruding from the cell surface around the junctions of the epithelial cell boundaries. Note the smaller size of the microvilli (m) covering most epithelial cells. Scale bars: 1 mm (a); 1 mm (b); 100 μm (c); 20 μm (d); 2 μm (e); 5 μm (f).

**FIGURE 5:**
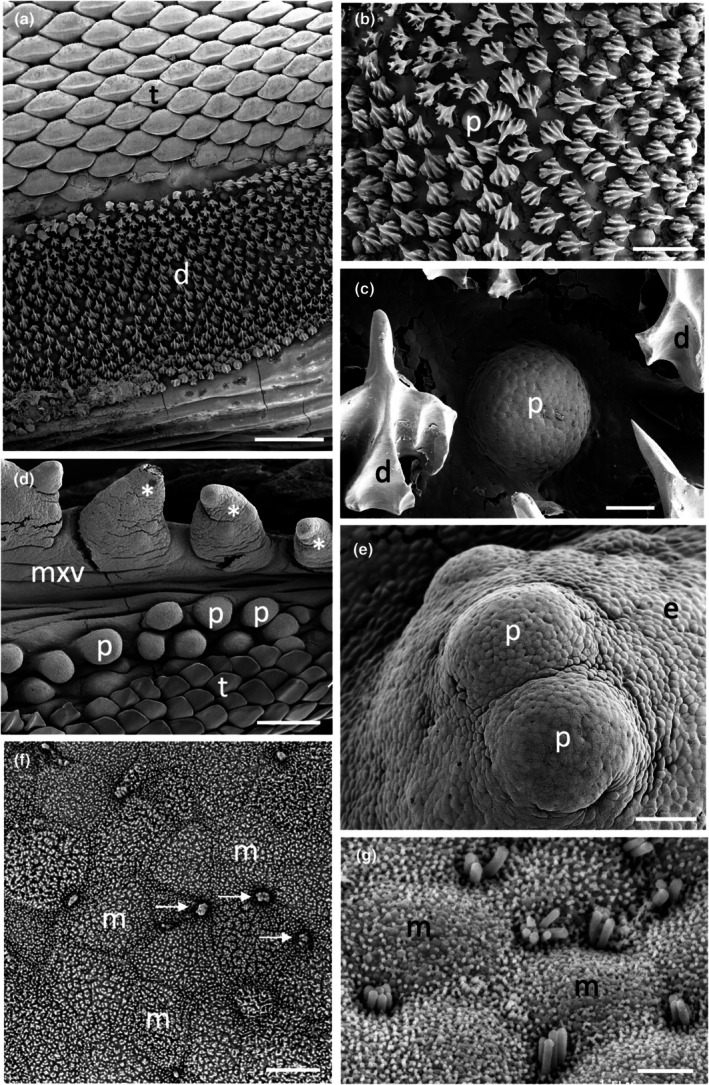
Taste papillae in the Eastern fiddler ray, *Trygonorrhina fasciata* viewed using scanning electron microscopy. (a) Low power of the mandibulary oral valve showing the packing of the teeth (t) and denticles (d). (b) Lower power of the central ventral pharynx showing a raised papilla amongst an array of denticles. (c) High power of a taste papilla (p) surrounded by five denticles (d). (d) Lower magnification of the maxillary oral valve (mxv) showing a row of finger‐like projections (asterisks) overlying a row of taste papillae (p) and a hexagonal array of teeth (t). (e) A series of three taste papillae (p) projecting from the lateral surface of a gill arch. (f) Small clumps of microvilli (arrows) protruding from the junctions of several epithelial cells situated at the crest of a taste papilla. (g) High power of the clumps of larger microvilli in a nearby region to (f), which may represent solitary chemosensory cells. e, epithelium; m, small microvilli. Scale bars: 1 mm (a); 200 μm (b); 20 μm (c); 200 μm (d); 50 μm (e); 3 μm (f); 2 μm (g).

The morphology of the taste papillae and denticles is described in two species of guitarfishes (Trygonorrhinidae), *Aptychotrema rostrata* and *Trygonorrhina fasciata* (Figures 4 and 5). The maxillary oral valve of the eastern shovelnose ray, *A. rostrata*, is a lappet with a bifurcated edge. On its lateral edges are a series of finger‐like projections, which have between one and four tips. Anterior to this finger‐like margin is an accumulation of Type I papillae, which taper at their lateral margins (Figure 4a). The most extreme protrusion of each bifurcation is densely covered in denticles with no finger‐like projections. The mandibular oral valve is flat with a linear distribution of three to four rows of papillae (155 ± 4 μm (*n* = 46) in diameter), lying directly posterior to the jaw (Figure 4b). Denticles are densely distributed all over the oropharyngeal region and gill arches (Figure 4c). Type II papillae (146 ± 9 μm (*n* = 11) in diameter), which protrude a lesser distance from the epithelium, are found in the oropharyngeal region (Figure 4d, Table 2). The oropharyngeal cavity is flat with no ridges, although large papilla‐like projections lie on the lateral margins of the flattened gill bars, which are devoid of denticles and have numerous papillae over their surface. Large and small microvilli (~0.3 μm in diameter and ~0.7 μm tall and ~0.1 μm in diameter and ~0.6 μm tall) not only project in small groups from various points (covering ~1.5% of the apical papillae tips, Figure 4e) but also project from the flat squamous epithelium (Figure 4f, Table 2).

The maxillary oral valve of the eastern fiddler ray, *T. fasciata*, is a bifurcated lobe projecting posteriorly from the jaw, the edge of which bears a series of finger‐like projections that may have one or up to three tips. Slightly anterior to this finger‐like margin are two to three rows of small Type I papillae, after which is a dense arrangement of denticles (Figure 5). The most extreme protrusion of each bifurcation is also densely covered in denticles. The mandibulary valve is flat with roughly 500 Type I papillae per cm^2^ distributed amongst numerous denticles (Figure 5a). Denticles are densely distributed all over the oropharyngeal region, although a clear boundary is formed just in front of the gill bars, which are devoid of denticles. Type II papillae are found in the oropharyngeal region (104 ± 0.4 μm in diameter, *n* = 8) (Table 2). The oropharyngeal cavity is flat with no ridges, although on the lateral margins of the flattened gill bars there are large bulbous projections that have three to five small papillae over their surface (Figure 5b,c). Nodular bulbous microvilli (~1 μm in diameter) project from various points all over the tips of papillae, covering ~1.6% of the papilla surface (Figure 5e, Table 2). Similar microvilli (~0.3 μm in diameter and ~0.4–1.3 μm tall) that resemble solitary chemosensory cells are also seen on the flat epithelium (Figure 5f,g) beside the papillae.

#### 3.1.2 Habitat, diet, and distribution of taste papillae

The eastern shovelnose ray, *A. rostrata*, is a benthic species commonly found in the shallow parts of estuaries and off beaches. The eastern fiddler ray, *T. fasciata* is also a benthic, shallow water species (Last & Stevens, 2009). Both feed predominantly on crustaceans (shrimps and crabs) and occasionally teleost fishes and cephalopods (Last & Stevens, 2009, Table 1). We were unable to observe *T. fasciata* feed, however, *A. rostrata* would swim closely over the surface of the substrate and upon finding a prey item, would suck it into the mouth consuming the item whole, head first. On some occasions, it would begin ‘chomping’ and manipulating the prey before discarding any hard exoskeletons (minus any flesh). Taste papillae are found on the maxillary and mandibular oral valves of each species. The maxillary oral valve of *A. rostrata* has ~13 papillae on finger‐like projections along each of the lateral most sections of its free edge and a cluster of ~620 papillae set back from the edge, which taper at the lateral most regions of the valve. The mandibular oral valve has a linear distribution of ~680 papillae between three and four deep along its length at a density of 2,140 cm^−2^ and ~190 papillae distributed amongst denticles, which occur at a density of 90 cm^−2^. The maxillary oral valve of *T. fasciata* has ~24 papillae on finger‐like projections along each of the lateral most sections of its free edge and a series of ~190 short papillae between two and three deep set back from the edge. The mandibular oral valve has a band of ~310 papillae at a density of 510 papillae cm^−2^ distributed amongst dense denticles (counted in one animal of 180 mm disc width, 189 mm disc length, 416 mm total length).

**FIGURE 6:**
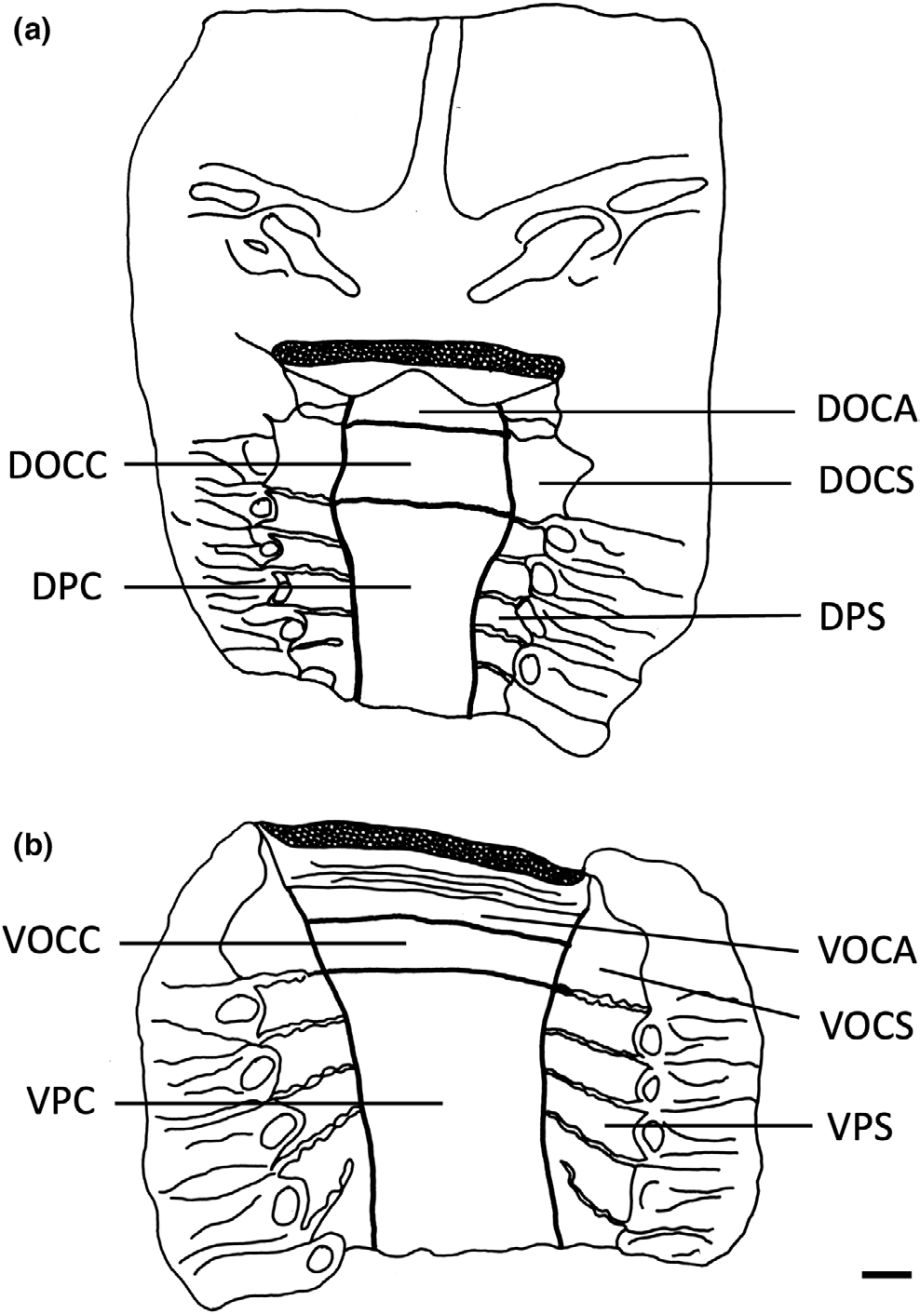
Mean oral papillae densities per cm^2^ ± SE for the various oropharyngeal regions of *Trygonorrhina fasciata* (*n* = 2, DW 180‐189 mm, DL 189‐196 mm, TL 416‐435 mm) in the maxilla (a) and mandible (b). Dots represent prominent taste papillae observed at the level of the light microscope. See Figure 6 for other abbreviations. Maxillary and mandibulary oral valves are not shown. Scale bars: 1.0 cm (a, b).

**FIGURE 7:**
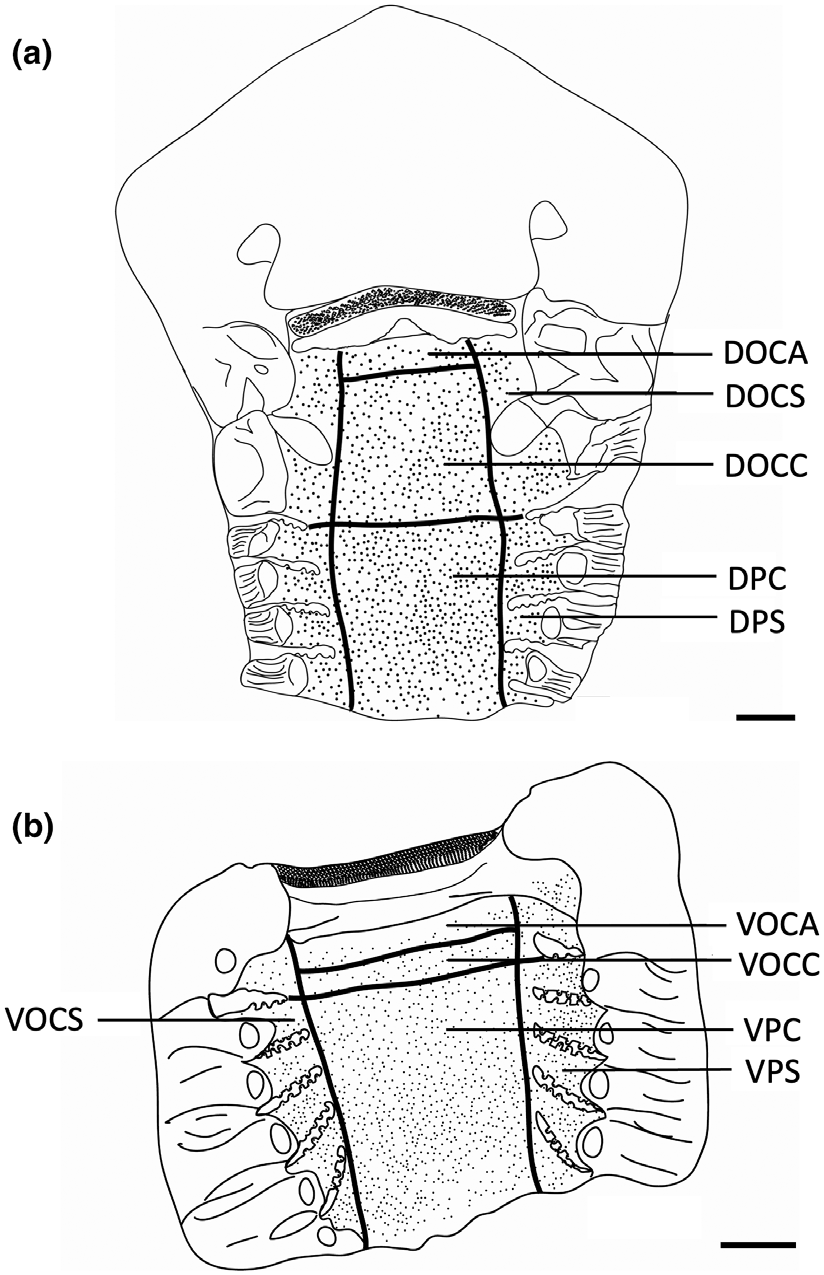
Mean oral papillae densities per cm^2^ ± SE for the various oropharyngeal regions of *Aptychotrema rostrata* (*n* = 1, TL 750 mm) in the maxilla (a) and mandible (b). DOCA, dorsal oral cavity anterior; DOCC, dorsal oral cavity centre; DOCS, dorsal oral cavity sides; DPC, dorsal pharynx centre; DPS, dorsal pharynx sides; VOCA, ventral oral cavity anterior; VOCC, ventral oral cavity centre; VOCS, ventral oral cavity sides; VPC, ventral pharynx centre; VPS, ventral pharynx sides. Dots represent prominent taste papillae observed at the level of the light microscope. Maxillary and mandibulary oral valves are not shown. Scale bars: 1.0 cm (a, b).

The teeth are small and densely arranged into a hard surface ideal for crushing hard‐bodied prey. Papilla density within the flat, central oropharyngeal cavity ranged from 130 to 160 papillae cm^−2^ and 110–160 papillae cm^−2^ in the dorsal and ventral regions in *A. rostrata*, respectively, with the lowest densities on the sides of the oral cavity and pharynx (Figure 6, Table 3). This species possesses a total of ~8,630 taste papillae. *T. fasciata* has considerably higher densities in the central regions of the upper and lower palate ranging from 114 to 159 papillae cm^−2^ and 100–138 papillae cm^−2^ in dorsal and ventral regions, respectively, with slightly higher densities on each side (Figure 7; Table 3) and a total number of 4,015 papillae located amongst very densely packed denticles. The gill arches of both species have large round projections covered with three to five papillae on the lateral most edges, elevating papillae into the region of water flow before it travels over the gill filaments.

### 3.2 Stingrays (Dasyatidae)

#### 3.2.1 Morphology of taste papillae

**FIGURE 8:**
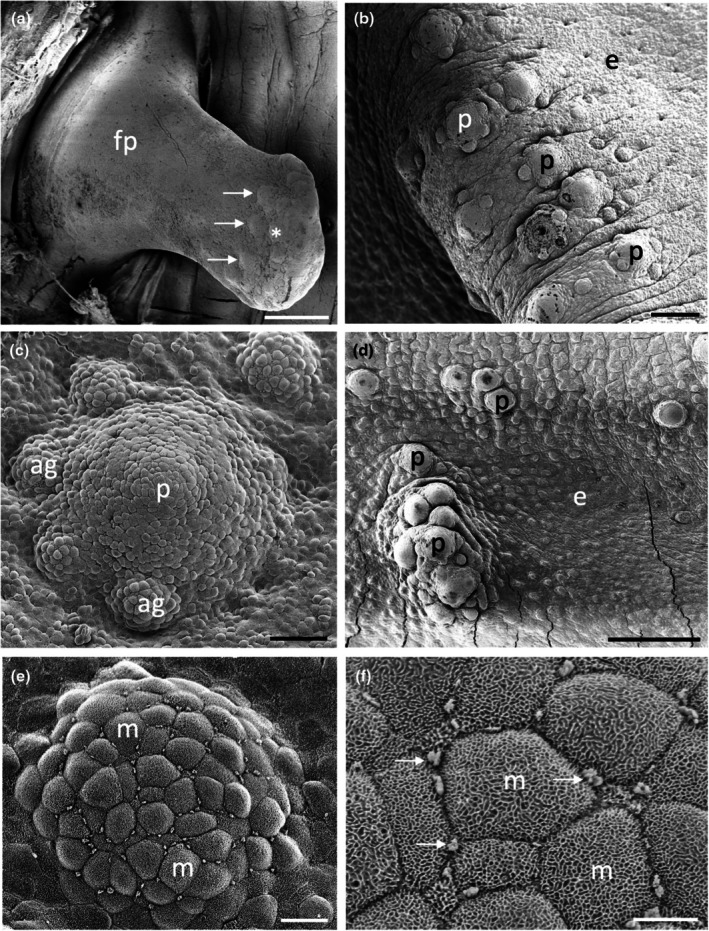
Taste papillae in the Estuary stingray, *Hemitrygon fluviorum* viewed using scanning electron microscopy. (a) Low power of a finger‐like projection (fp) in the anterior, ventral oral cavity showing a row of larger taste papillae (arrows) surrounding smaller papillae (asterisk) towards the tip. (b) Low magnification of a ridge on the side of the ventral oral cavity showing raised taste papillae (p) protruding from the surface of the epithelium (e). Note up to five small aggregations of epithelial cells surround the base of each papilla. (c) Higher magnification of a taste papilla (p) showing five circular epithelial cell aggregations (ag) at its base. (d) Clumps of taste papillae (p) which occur over many areas of the gill arches. (e) High power of the circular epithelial cell aggregations seen in (c). (f) High power of the epithelial cells in (e) showing tufts of longer microvilli (arrows) protruding from regions along the junctions of opposing epithelial cells, which may represent solitary chemosensory cells. e, epithelium. Scale bars: 0.5 mm (a); 200 μm (b); 50 μm (c); 0.5 mm (d); 10 μm (e); 5 μm (f).

**FIGURE 9:**
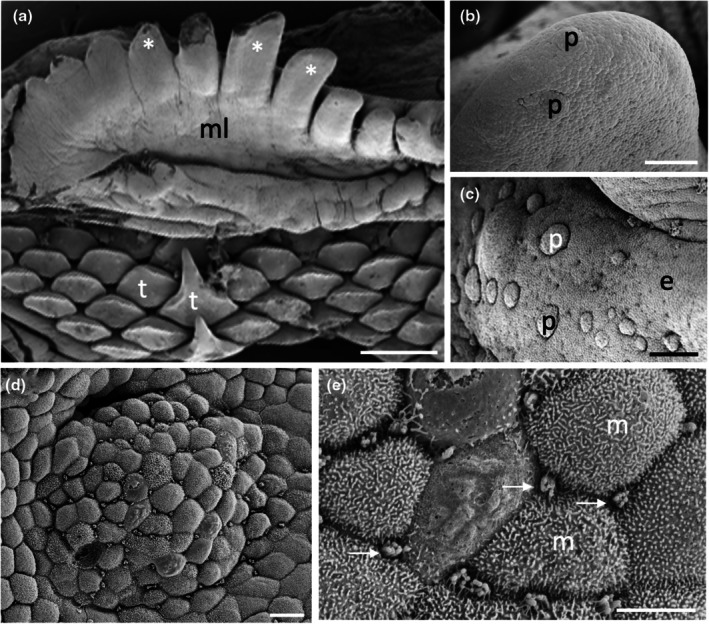
Taste papillae in the Blue‐spotted stingray, *Neotrygon kuhlii* viewed using scanning electron microscopy. (a) The maxillary lobe (mL) with finger‐like projections (asterisks) along the free edge, overlying rows of teeth (t). (b) High power of the apical tip of one of the finger‐like projections in (a) showing a number of taste papillae (p) over the surface. (c) Ridge at the centre of the dorsal oral cavity with several taste papillae protruding from the epithelium (e). (d) Higher power of a small papilla from the central dorsal pharynx. (e) Magnified view of the tip of the small taste papillae in (d) showing solitary chemosensory microvilli (arrows) amongst numerous epithelial cells covered in small microvilli (m). Scale bars: 1 mm (a); 100 μm (b); 100 μm (c); 10 μm (d); 5 μm (e).

**FIGURE 10:**
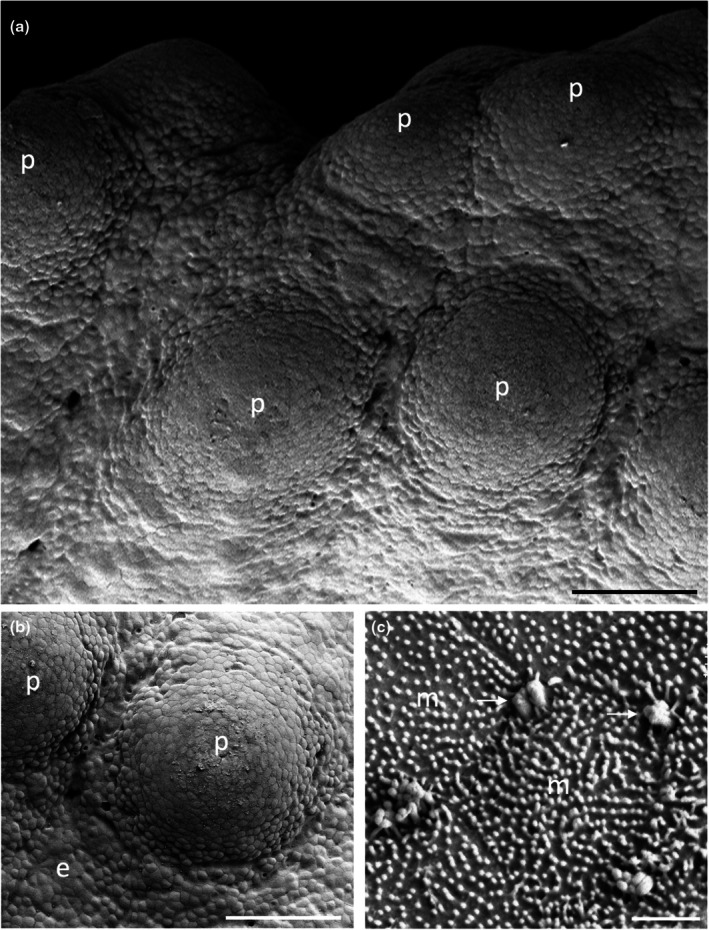
Taste papillae in the Blue‐spotted fantail ray, *Taeniura lymma* viewed using scanning electron microscopy. (a) Low power of a ridge on the anterior region of the dorsal oral cavity showing multiple taste papillae (p). (b) Taste papillae (p) protruding from the epithelial surface (e) in the ventral oral cavity. (c) High power of the epithelial surface covered in small microvilli (m) with several clumps of longer microvilli (arrows) thought to be solitary chemosensory receptors. Scale bars: 100 μm (a); 100 μm (b); 2 μm (c).

The morphology of taste papillae is described for three species of dasyatids that were sampled: the estuary stingray, *Hemitrygon fluviorum*, the blue‐spotted stingray, *Neotrygon kuhlii*, and the blue‐spotted fantail ray, *Taeniura lymma* (Figures 8, 9 and 10). The oral valves of *H. fluviorum* appear as a singular maxillary lobe with a bifurcated edge and seven mandibular finger‐like projections (~0.7–1.4 mm in diameter), which become progressively smaller from the centre (5.6 mm tall) to the most lateral projection (1.2 mm tall) (Figure 8a,b). The free edge of the maxillary lobe is scalloped with no obvious papillae like those seen in the oropharyngeal cavity but does possess microvilli, which appear comparable in structure to solitary chemosensory cells. Small and large papillae (~30 μm and ~90 μm in diameter, respectively) are seen on these mandibular projections. The oropharyngeal cavity surface is not flat but has a series of ridges both dorsally and ventrally. These ridges have higher concentrations of papillae (159 ± 4 μm (*n* = 34) in diameter) than the surrounding flat epithelium. The majority of the papillae on these ridges are surrounded by up to five smaller papillae (56 ± 1 μm (*n* = 88) in diameter, Figure 8c,d). The flat regions of squamous epithelium spanning the region between these ridges contain individual or multiple groups of papillae comparable in structure and distribution to Type II taste buds of teleosts. Microvilli (~0.3 μm in diameter and ~0.4–1.3 μm tall) protrude regularly over the whole surface of the papilla but in much smaller groups, more like solitary chemosensory cells (Figure 8e,f) covering a total area of ~1.6% of the papilla surface area (Table 2). No structures comparable to Type III taste buds in pits were observed using SEM. Histology reveals the papillae are comprised of an area of differentiated cells arranged in an egg‐cup‐like formation of the basal lamina (Figure 3). These cells are elongated and may form the villous projections observed using SEM.

*N. kuhlii* has a singular maxillary lobe with a bifurcated edge lined with finger‐like projections, which contain papillae, and two mandibular finger‐like projections (1.4 mm in diameter and 4.6 mm tall) on either side of the central line (Figure 9a,b). The oropharyngeal cavity also has a series of ridges, like those of *H. fluviorum*, with higher concentrations of papillae (Figure 9c). The smaller papillae (62 ± 4 μm (*n* = 17) in diameter) not only surround larger papillae (192 ± 7 μm (*n* = 11) in diameter) but also occur individually on the ridges and the flat oropharyngeal epithelium (Figure 9d). Microvilli (~0.3 μm in diameter and ~0.5–0.9 μm tall) appear more like solitary chemosensory cells (Figures 9d,e) and protrude all over the papilla tip covering ~1.5% of the total papillar surface area (Table 2). No Type III taste buds were observed at the level of the SEM.

The oral valves of *T. lymma* are very similar to those of *N. kuhlii* with a maxillary lobe containing a bifurcated edge lined with finger‐like projections and two mandibular finger‐like projections (~1 mm in diameter and ~3 mm tall). High concentrations of papillae are found on a series of ridges in the oropharyngeal cavity and on the flat epithelium (Figure 10a,b). Papillae are 143 ± 6 μm (*n* = 12) in diameter (Figure 10b, Table 2) and microvilli (~0.3 μm in diameter and ~0.3–0.6 μm tall) appear more like solitary chemosensory cells and protrude all over the papilla tip, covering a total area of ~0.7% of the papilla surface area (Figure 10c, Table 2). No Type III taste buds were observed using SEM. Histological sections reveal a differentiated region of elongated cells protruding above the surface of the papilla epithelium (Figure 3).

#### 3.2.2 Habitat, diet and distribution of taste papillae

The estuary stingray, *H. fluviorum*, is a benthic species associated with sand flats, mangrove swamps, and estuaries, which feeds on crabs, polychaete worms, and other crustaceans (Last & Stevens, 2009). The blue‐spotted mask ray, *N. kuhlii*, is a benthic inshore species from the sand flats and feeds predominantly on polychaete worms and sometimes crustaceans and small fishes (Last & Stevens, 2009). The blue‐spotted fantail ray, *T. lymma*, is a benthic shallow coral reef species; although during the rising tide, it migrates to sandy flats and intertidal reefs to feed on molluscs, worms, and small crustaceans (Last & Stevens, 2009). In all species, the teeth are small and densely arranged into a hard surface ideal for crushing hard‐bodied prey.

**FIGURE 11:**
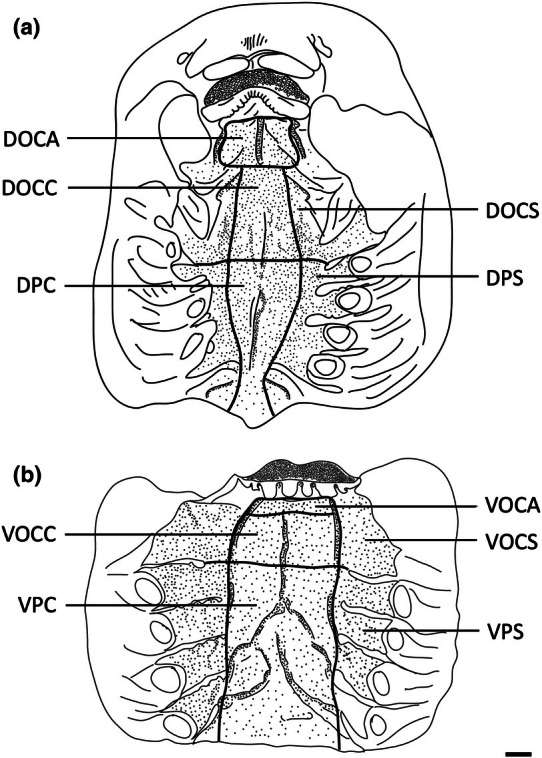
Mean oral papillae densities per cm^2^ ± SE for the various oropharyngeal regions of *Hemitrygon fluviorum* (*n* = 1, DW 383 mm, DL 353 mm, TL 918 mm) in the maxilla (a) and mandible (b). Dots represent prominent taste papillae observed at the level of the light microscope. See Figure 6 for other abbreviations. Maxillary and mandibulary oral valves are not shown. Scale bars: 1.0 cm (a, b).

**FIGURE 12:**
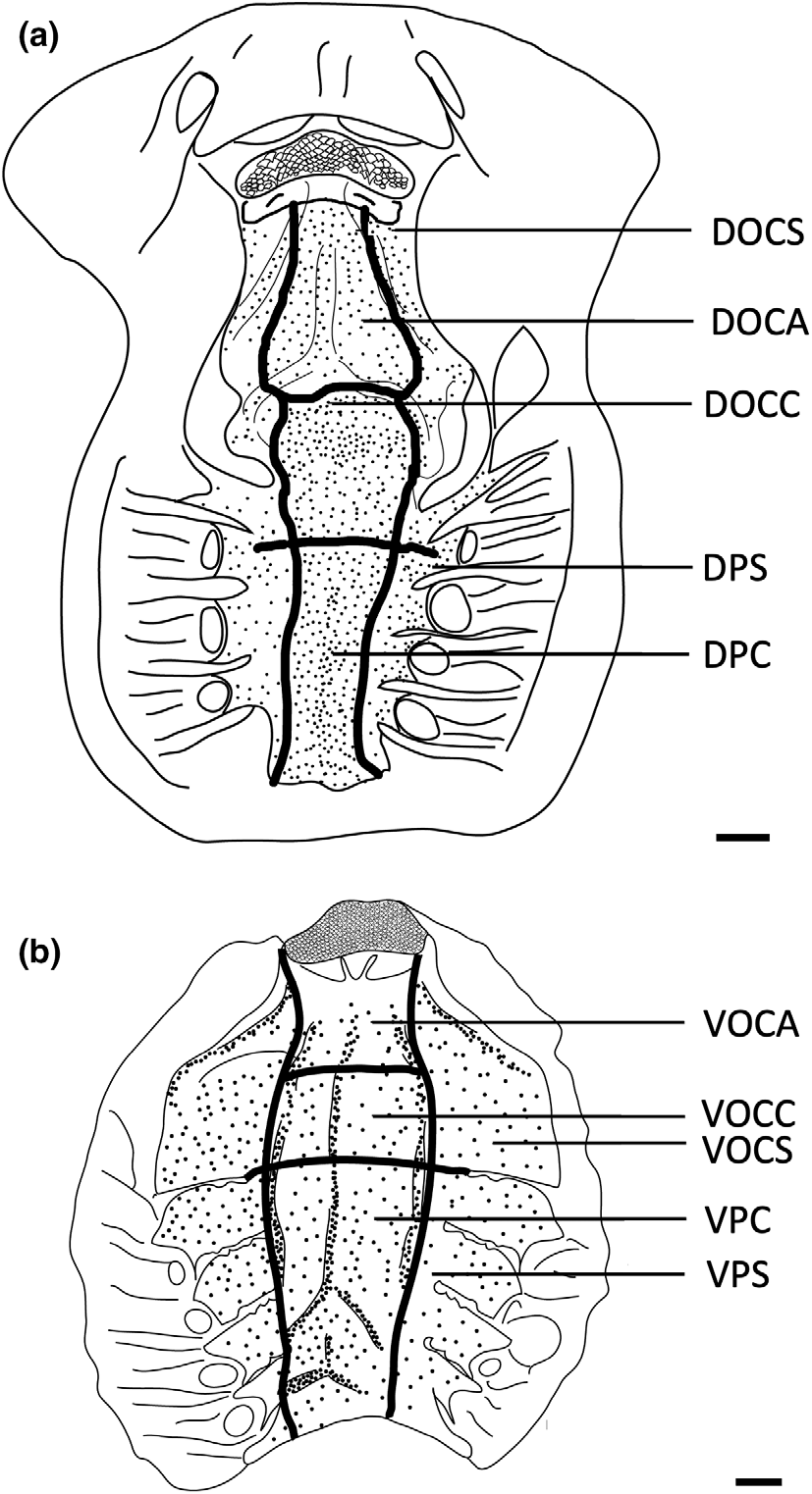
Mean oral papillae densities per cm^2^ ± SE for the various oropharyngeal regions of *Neotrygon kuhlii* (*n* = 1, DW 363 mm, DL 290 mm, TL unknown) in the maxilla (a) and mandible (b). Dots represent prominent taste papillae observed at the level of the light microscope. See Figure 6 for other abbreviations. Maxillary and mandibulary oral valves are not shown. Scale bars: 1.0 cm (a, b).

**FIGURE 13:**
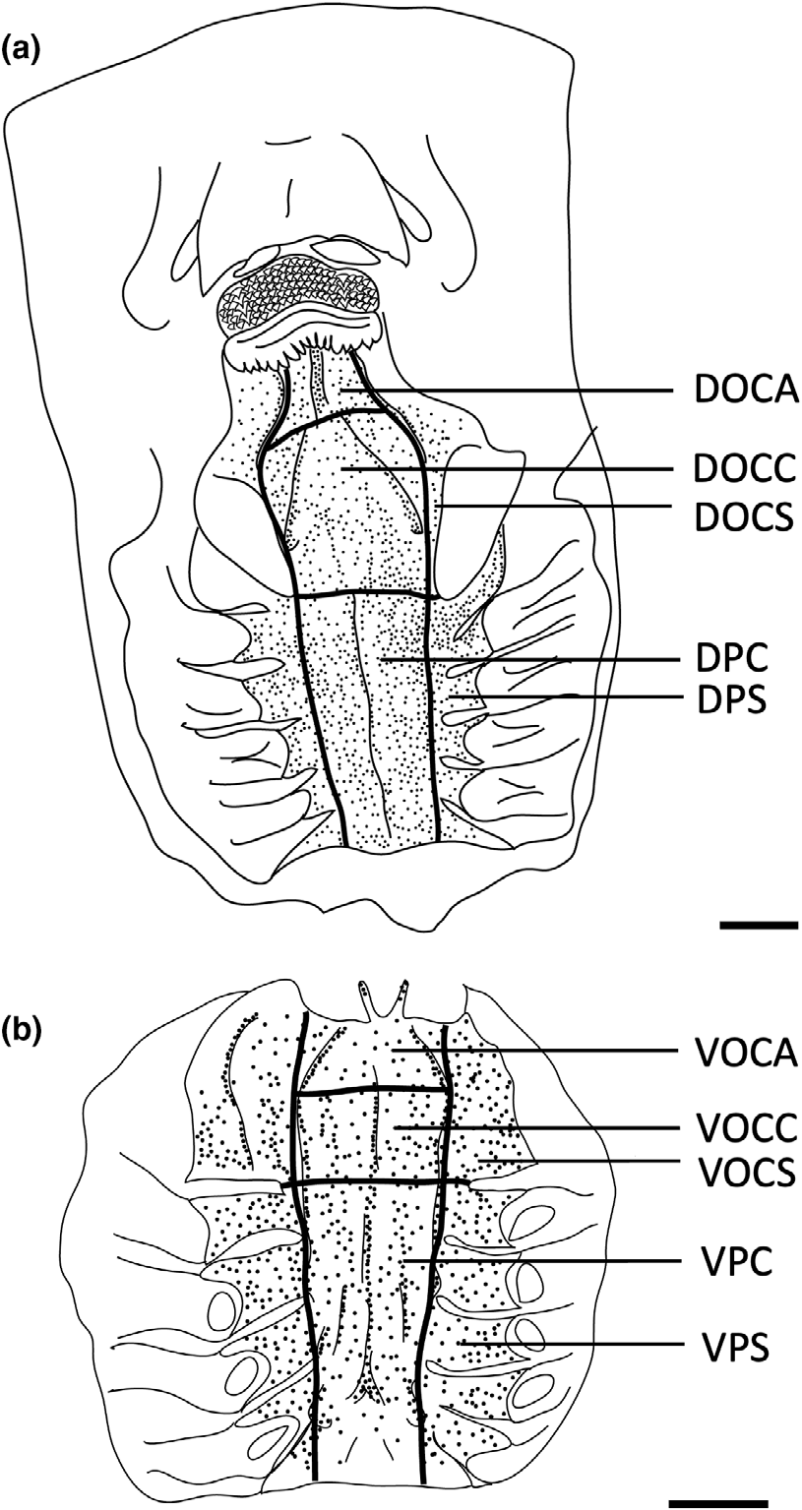
Mean oral papillae densities per cm^2^ ± SE for the various oropharyngeal regions of *Taeniura lymma* (*n* = 1, DW 169 mm, DL 184 mm, TL 418 mm) in the maxilla (a) and mandible (b). Dots represent prominent taste papillae observed at the level of the light microscope. See Figure 6 for other abbreviations. Maxillary and mandibulary oral valves are not shown. Scale bars: 1.0 cm (a, b).

Each of these species has a series of ridges within their oropharyngeal regions (Figures 11, 12, 13). There are three prominent ridges in the dorsal oral cavity; one positioned centrally with the other two on either side. In the posterior region of the oral cavity, there is a less pronounced ridge on either side of the cavity. These ridges originate near the most posterior point of the central anterior ridge and terminate on the anterior edge of the spiracle, which may be associated with hydrodynamic movement of water within the oral cavity. A similar series of ridges is seen on the ventral surface, although these ridges appear to protrude a lesser distance. Ridges on the ventral surface are also located in the pharyngeal region and appear to direct water flow over the gills. All of these ridges have dense accumulations of both large and small papillae around them. *H. fluviorum* has densities of ~2,560 (small) and 1,610 (large) papillae cm^−2^ along these ridges, totalling 9,343 and 5,878 papillae, respectively (Figure 11), whereas *N. kuhlii* has densities of ~3,430 (small) and 850 (large) papillae cm^−2^ along the ridges alone, totalling 4,550 and 1,130 papillae, respectively (Figure 12). In *T. lymma*, there is no significant difference in taste papilla size, with densities reaching 175 papillae cm^−2^ totalling ~1,210 on all the ridges (Figure 13).

Over the flat oropharyngeal epithelium, taste papillae densities are much lower. In *H. fluviorum*, densities range from 64 to 79 papillae cm^−2^ and 33–80 papillae cm^−2^ in dorsal and ventral (central) regions, respectively (Figure 11, Table 3). In *N. kuhlii*, the density of papillae varies between 47 and 81 papillae cm^−2^ and from 61 to 91 papillae cm^−2^ in dorsal and ventral (central) regions, respectively (Figure 12, Table 3). *T. lymma* appears to have a larger distribution of papillae on the dorsal surface with densities between 120 and 175 papillae cm^−2^ as opposed to the ventral surface, which has densities between 55 and 88 papillae cm^−2^ (Figure 13, Table 3). Total numbers of taste papillae on the flat epithelial surface vary between species, with 5,100 (of a total of 20,317) present in *H. fluviorum*, 4,150 (of a total of 9,834) in *N. kuhlii*, and 1,730 (of a total of 2,950) in *T. lymma* (Table 3).

### 3.3 Butterfly rays (Gymnuridae)

#### 3.3.1 Morphology of taste papillae

**FIGURE 14:**
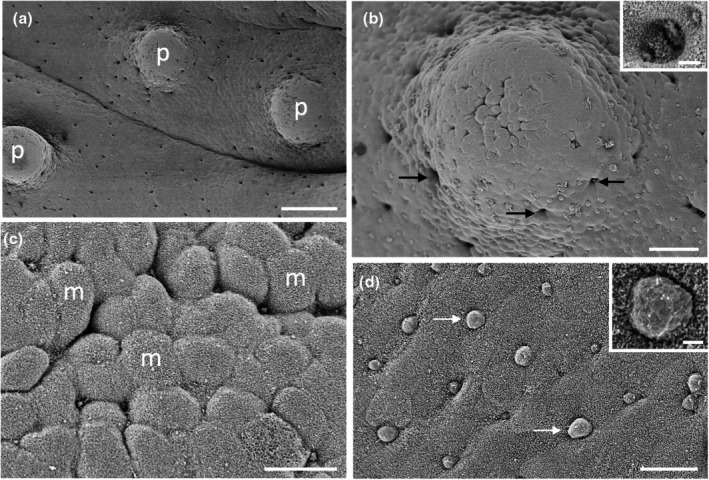
Taste papillae in the Australian butterfly ray, *Gymnura australis* viewed using scanning electron microscopy. (a) Low magnification of papillae (p) on the lateral side of the ventral oral cavity. (b) Higher power of a taste papilla showing numerous pores around the base of the papilla (arrows). Inset shows a high power of one of these pores with numerous microvilli (m) protruding around the edges of the pore. (c) Differentiated region of epithelial cells and depressions on the apical tip of a papilla. (d) The array of bulbous nodules (arrows) over the side of the dorsal oral cavity. Inset shows a high power of one of these nodules. Scale bars: 200 μm (a); 50 μm (b); 2 μm (inset in (b)); 10 μm (c); 20 μm (d); 1 μm (Inset in (d)).

Only one species of Gymnuridae was sampled, the Australian butterfly ray, *Gymnura australis*. The oral valves of *G. australis* consist of a singular maxillary lobe with a bifurcated edge lined with a series of finger‐like projections and a single mandibular lobe also with finger‐like projections along the margin. The oropharyngeal cavity is flat like that found in the Trygonorrhinidae, and papillae are distributed evenly over this surface (Figure 14a,b). Papillae are 220 ± 6 μm (*n* = 13) in diameter and have a series of depressions over their tip covering a total area of ~0.7% of the papilla surface (Figure 14b, Table 2). High concentrations of depressions were observed over the oropharyngeal surface (Figure 14b), with the taste papillae resembling Type III taste buds. Various other bulbous projections were also observed over many parts of the oropharyngeal region (Figure 14d).

#### 3.3.2 Habitat, diet, and distribution of taste papillae

**FIGURE 15:**
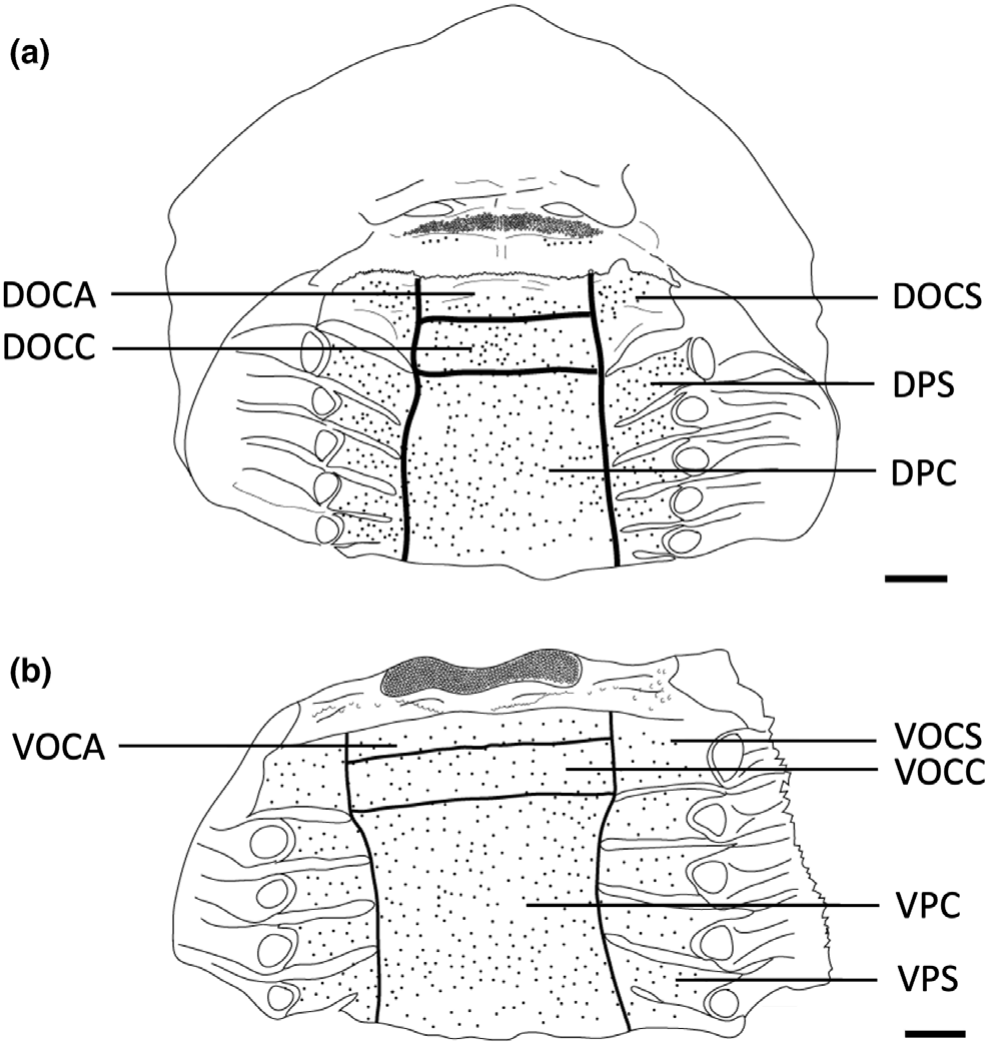
Mean oral papillae densities per cm^2^ ± SE for the various oropharyngeal regions of *Gymnura australis* (*n* = 2, DW 399‐475 mm, DL 210‐252 mm, TL 296‐330 mm) in the maxilla (a) and mandible (b). Dots represent prominent taste papillae observed at the level of the light microscope. See Figure 6 for other abbreviations. Maxillary and mandibulary oral valves are not shown. Scale bars: 1.0 cm (a, b).

The Australian butterfly ray, *G. australis*, is a benthic species found on sandy flats but is also caught regularly by prawn trawlers. It feeds predominantly on teleost fishes but its diet may also include crustaceans and cephalopods (Last & Stevens, 2009). The teeth are small and densely arranged into a hard surface ideal for crushing hard‐bodied prey. There are a total of 2,119 papillae distributed relatively evenly throughout the oropharyngeal region (Figure 15) with densities of taste papillae of between 50 and 65 papillae cm^−2^ and between 65 and 73 p,apillae cm^−2^ within the dorsal and ventral regions of the oropharynx, respectively, including the sides (Table 3).

### 3.4 Wobbegong sharks (Orectolobidae)

#### 3.4.1 Morphology of taste papillae

Two species of orectolobid sharks were sampled: the spotted wobbegong shark, *Orectolobus maculatus*, and the ornate wobbegong shark, *Orectolobus ornatus*. Low numbers of circular Type II papillae were observed all over the oral cavity, pharynx, and gill bars in *O. maculatus*, with each taste papilla possessing a region of differentiated cells at its apical tip. Papilla diameter of the two larger specimens was significantly larger at the 5% significance level than that of the two smaller individuals, and so two groups were formed with the specimens of this species. For the two smaller individuals, papillae diameters were measured to be 254 ± 7 μm (*n* = 30), while papilla diameter increased to 359 ± 13 μm (*n* = 31) in the larger two specimens (Table 2).

**FIGURE 16:**
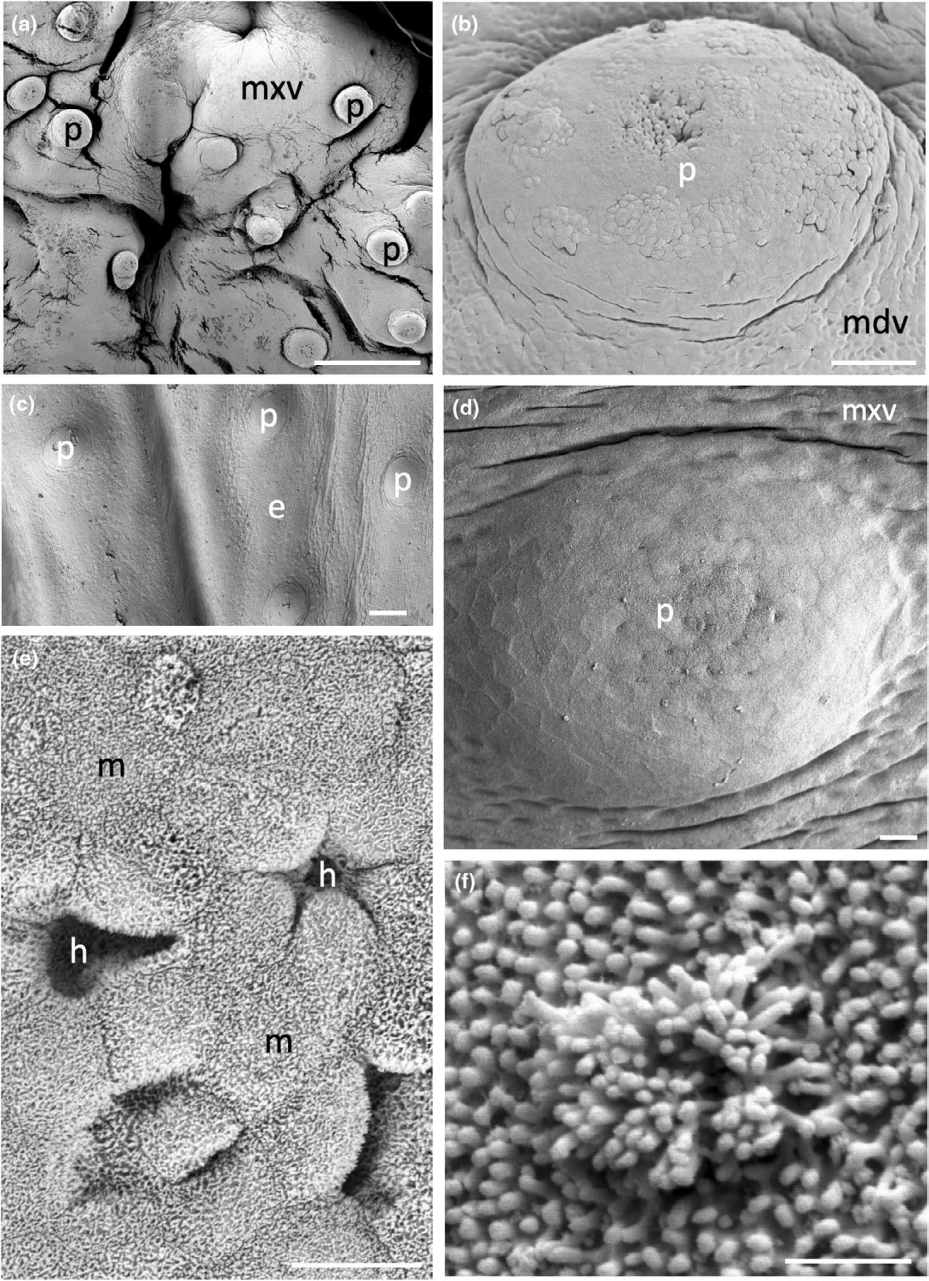
Taste papillae in the Spotted wobbegong shark, *Orectolobus maculatus* viewed using scanning electron microscopy. (a) A series of raised taste papillae (p) over the maxillary valve (mxv). (b) A raised taste papilla (p) protruding from the surface of the mandibular valve (mdv). Note the differentiated region in the centre. (c) Low power of the papillae (p) over the lateral region of the dorsal oral cavity. (d) A taste papilla (p) from the maxillary valve (mxv). (e) Apical tip of a papilla situated within the central dorsal oral cavity. Note the deep holes (h) at the cellular junctions and the dense mat of small microvilli (m) covering the epithelial cells. (f) High power of a clump of long microvilli amongst a mat of smaller microvilli at the apical tip of a papilla on the maxillary valve. Scale bars: 1 mm (a); 100 μm (b); 100 μm (c); 20 μm (d); 10 μm (e); 1 μm (f).

**FIGURE 17:**
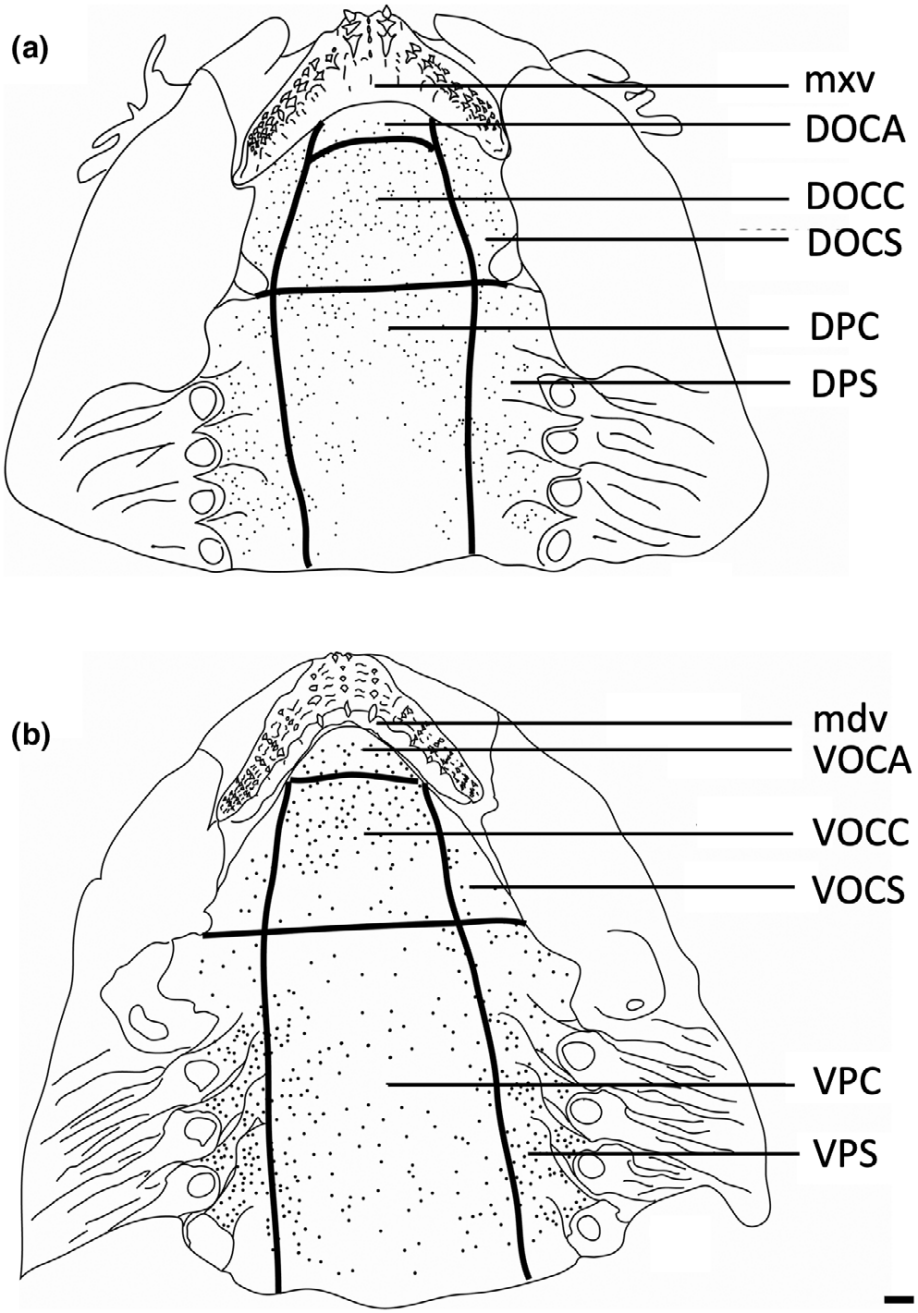
Mean oral papillae densities per cm^2^ ± SE for the various oropharyngeal regions of *Orectolobus maculatus* (*n* = 4, TL 674–1172 mm) in the maxilla (a) and mandible (b). Maxillary oral valve (mxv), mandibulary valve (mdv). Dots represent prominent taste papillae observed at the level of the light microscope. See Figure 6 for other abbreviations. Scale bars: 1.0 cm (a, b).

**FIGURE 18:**
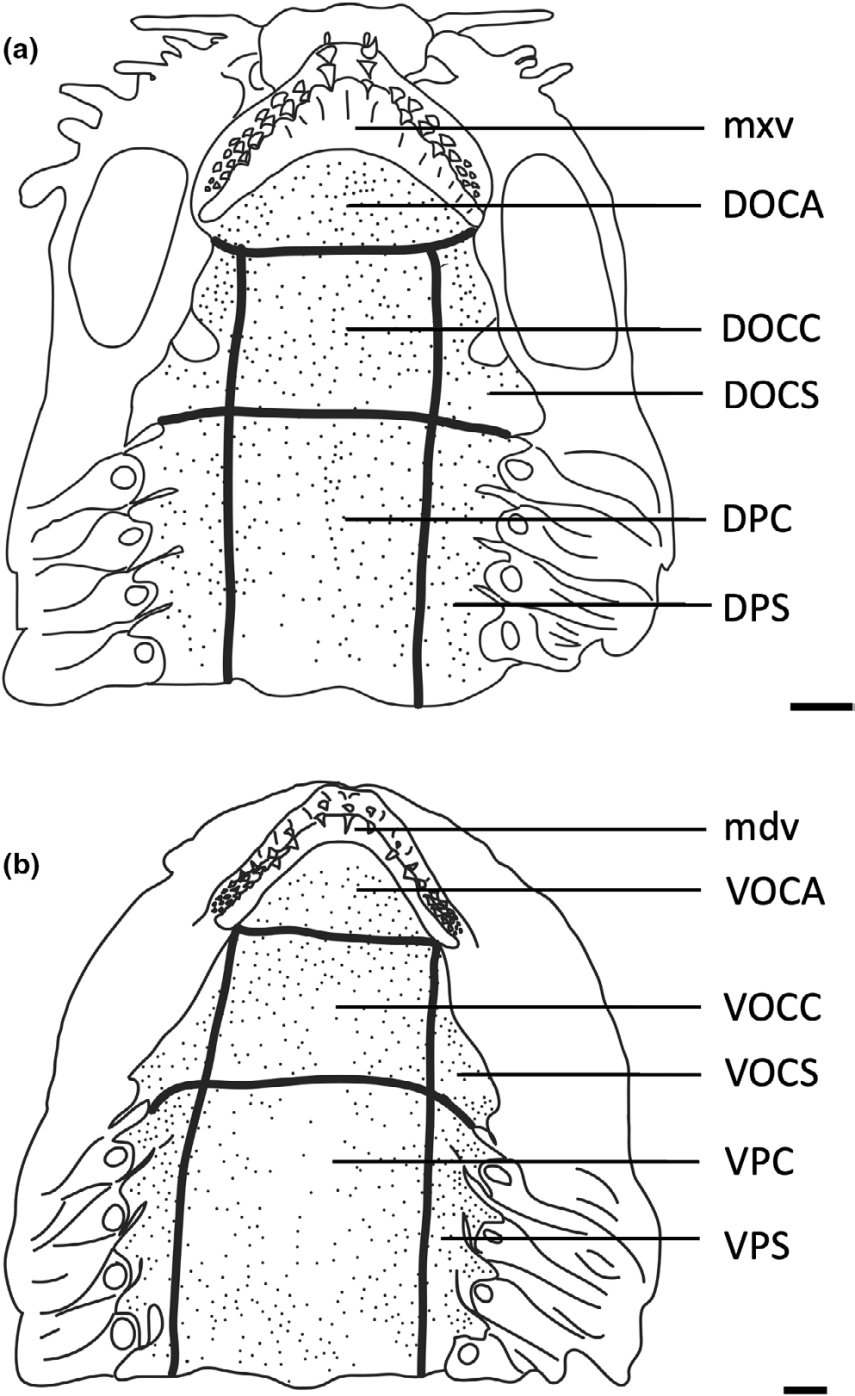
Mean oral papillae densities per cm^2^ ± SE for the various oropharyngeal regions of *Orectolobus ornatus* (*n* = 3, TL 507‐629 mm) in the maxilla (a) and mandible (b). Maxillary oral valve (mxv), mandibulary valve (mdv). Dots represent prominent taste papillae observed at the level of the light microscope. See Figure 6 for other abbreviations. Scale bars: 1.0 cm (a, b).

Microvilli either protruded within depressions at the apical tip, or protruded above the epithelial surface covering ~0.4% of the papilla surface area. Higher concentrations of papillae were located on the oral valves directly behind the jaws (Figure 16a,b,d). These papillae appear to protrude further above the epithelial surface than those of the oropharyngeal cavity and are considered comparable to Type I taste papillae, although they lacked a prominent basal depression. The maxillary oral valve is U‐shaped and tapers at the edges. It protrudes some distance behind the upper jaw and is fleshy with a clearly defined margin raised above the surface of the epithelium within the dorsal oral cavity. In comparison, the mandibular oral valve protrudes much less and lies almost in line with the most posterior row of teeth in the lower jaw (Figures 17 and 18). The mandibular oral valve is also fleshy with an elevated and prominent margin; yet this does not overlap the oral cavity margin, as is the case for the maxillary oral valve, thereby creating a small gap.

The oropharyngeal surfaces are relatively flat with only a slightly concave dorsal surface and a slightly convex ventral surface. When the mouth is closed, opposing surfaces could touch with no significant cavities being created between them. Both the oral cavity and pharynx are very broad due to the dorso‐ventrally flattened shape of the head and, as a result, the gill bars are short in comparison to those of non‐dorso‐ventrally flattened species.

No obvious differences were observed between *O. ornatus* and *O. maculatus*. The surface structure of the stratified squamous epithelial cells is covered in dense patterns of microvilli and evenly distributed circular, Type II papillae with a diameter of 256 ± 21 μm (*n* = 14) observed all over the oral cavity, pharynx, and gill bars (Figure 16d,e; Table 2). Each papilla has a region of differentiated cells covering ~0.5% of its apical tip, where the larger microvilli of taste bud cells can also be observed projecting above the surface of the epithelial surface in multiple small groups (Figure 16e,f).

#### 3.4.2 Habitat, diet and distribution of taste papillae

The spotted wobbegong, *O. maculatus* is often found sheltering in caves during the day and then moves into shallow water on reefs and sand flats to feed nocturnally, predominately on reef fishes, octopi and often crabs (Last & Stevens, 2009). The ornate wobbegong, *O. ornatus* is associated with shallow water among coral heads and feeds predominantly on teleost fishes (Last & Stevens, 2009). During observations of feeding events, it was noted that they adopt a ‘sit and wait’ ambush strategy before eliciting a rapid suction feeding mechanism to engulf prey. Prey, generally when located directly overhead, almost instantaneously disappear as they are sucked into the oropharyngeal cavity, with the widest part (generally the head) of the prey engulfed first, and consumed whole (small prey). The discovery of whole fish found in the stomachs of dissected specimens also supports this feeding mechanism. After prey consumption, the wobbegong rises up on its pectoral fins and elevates the head. A degree of ‘chomping’ may occur during this time, possibly to flush the oral cavity and/or assist the easy passage of food down the oesophagus. Larger prey items may first be grasped between the powerful jaws with the aid of long, sharp, slender teeth, before being broken up and consumed.

Taste papillae are distributed throughout the entire oropharyngeal cavity (Figures 17 and 18). Topographical analyses reveal taste papilla densities vary between central regions and the sides of the oropharynx, with densities between 4 and 14 papillae cm^−2^ and between 5 and 11 papillae cm^−2^ in the dorsal and ventral (central) regions in *O. maculatus*, respectively. These compare to between 18 and 21 papillae cm^−2^ and between 15 and 18 papillae on the sides of the oropharyngeal cavity in dorsal and ventral regions, respectively. A similar arrangement occurs in *O. ornatus*, but with papillae densities reaching 40 and 38 papillae cm^−2^ in the dorsal and ventral oropharynx, respectively (Figures 17 and 18; Table 3). The oral valves contain the highest densities of taste papillae, with *O. maculatus* possessing 31 and 33 cm^−2^ in the maxillary and mandibulary oral valves, respectively, and *O. ornatus* possessing 84 and 61 cm^−2^ in the maxillary and mandibulary oral valves, respectively. Both species of wobbegong sharks have similar total numbers of taste papillae (*O. maculatus*; 1,916 ± 356; *O. ornatus*: 1,993 ± 149) (Table 2).

### 3.5 Carpetsharks (Hemiscylliidae)

#### 3.5.1 Morphology of taste papillae

**FIGURE 19:**
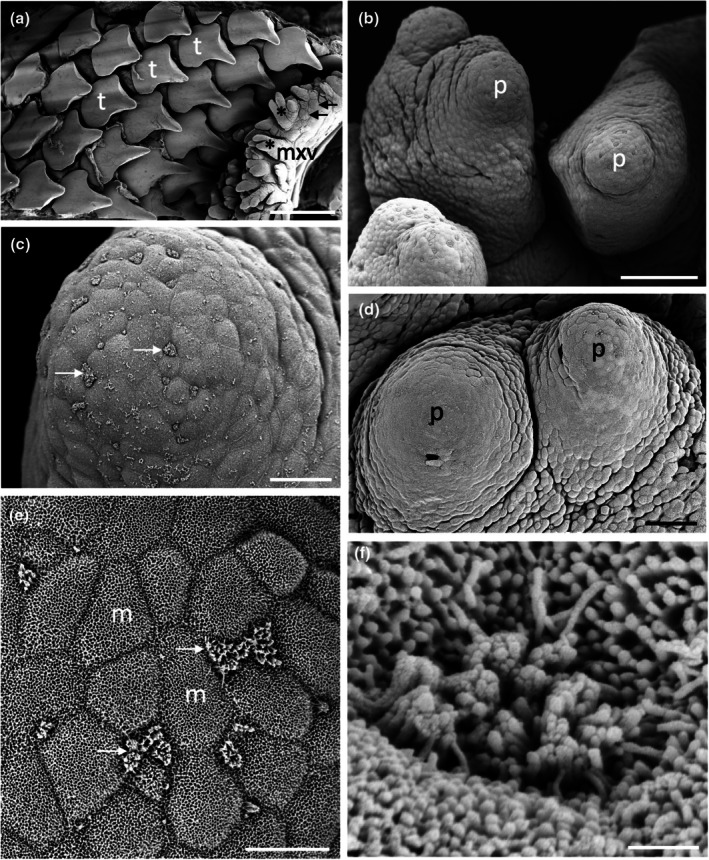
Taste papillae in the Epaulette shark, *Hemiscyllium ocellatum* viewed using scanning electron microscopy. (a) Finger‐like projections (asterisks) adjacent to a number of papillae (p) (arrows) on the maxillary oral valve (mxv). (b) Higher power of taste papillae (p) along the maxillary oral valve. (c) The apical tip of one of maxillary oral valve papilla showing clumps of large microvilli (arrows). (d) A group of taste papillae (p) from the anterior region of the ventral oral cavity. (e) Clumps of long microvilli (arrows) in the apical tip of a taste papilla. (f) High power of the long microvilli protruding from a small epithelial depression in the apical surface of a papilla. Scale bars: 1 mm (a); 100 μm (b); 20 μm (c); 50 μm (d); 10 μm (e); 1 μm (f).

Only one species of the Hemiscylliidae was sampled: the epaulette shark, *Hemiscyllium ocellatum*. The oral valves of *H. ocellatum* are U‐shaped tapering at the edges and comprised of numerous finger‐like projections of various heights, some of which are fused together with others occurring individually (Figure 19a–c). These papillae appear more comparable to Type I taste papillae. The oral valves appear to be more flap‐like and movable than the more rigid valves of the Orectolobidae, with their papillae protruding at various angles from the supporting epithelium. Microvilli protrude from the majority of the papilla tips and are not limited to the central apical region, and can be either adjacent to each other or separated by non‐sensory cells (Figure 19e,f).

Papillae are found in the oropharyngeal cavity either individually or in pairs (Figure 19d) protruding to various heights above the epithelium. Taste papillae are 175 ± 6 μm (*n* = 130) in diameter and their bases are level with the surrounding epithelium (Table 2). These papillae are comparable to Type II taste buds. Microvilli protrude in groups over the apical surface of the papillae, although microvilli are more limited to the central apical region covering ~1.6% of the papillar surface area (Figure 19f). No Type III taste buds were observed in this species at the level of the SEM. Histology reveals individual and groups of elongated cells protruding above the epithelial surface of the papilla (Figure 3e). Interestingly, these cells do not appear to extend to the basal lamina, as with other vertebrate taste buds, but lie above the basal lamina.

#### 3.5.2 Habitat, diet and distribution of taste papillae

The epaulette shark, *H. ocellatum*, is a benthic, shallow water reef species that feeds opportunistically, predominantly on worms and crabs, but also on shrimps, small fishes, and amphipods (Last & Stevens, 2009). Feeding observations reveal that they are suction‐feeders and often search for food items by moving their heads from side to side against the substrate. Upon finding a prey item, it is inhaled, and the animal rises up on its pectoral fins and puts the head down, forming an arch. Chomping of the item may follow for several minutes, with discarded pieces of tissue, such as exoskeleton, either spat back out from the mouth or released through the gills. Items that are considered large for the animal may often be held between the jaws while the head shakes from side to side as the item is broken into smaller pieces. The teeth are triangular, yet small, with broad bases and are densely orientated in rows that form a hard flat surface to enable hard‐bodied prey to be crushed.

**FIGURE 20:**
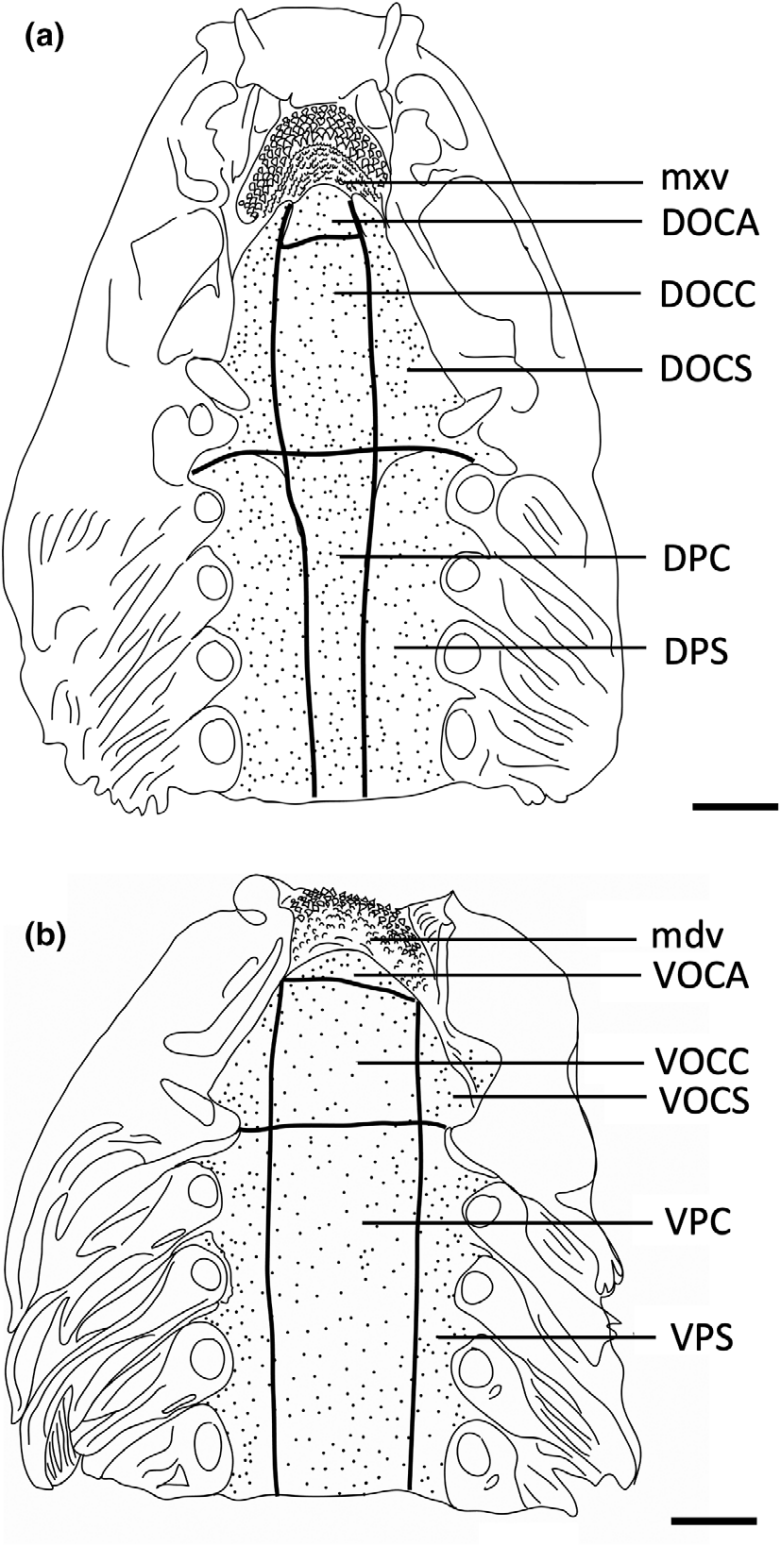
Mean oral papillae densities per cm^2^ ± SE for the various oropharyngeal regions of *Hemiscyllium ocellatum* (*n* = 4, TL 585‐655 mm) in the maxilla (a) and mandible (b). Maxillary oral valve (mxv). Mandibulary oral valve (mdv). Dots represent prominent taste papillae observed at the level of the light microscope. See Figure 6 for other abbreviations. Scale bars: 1.0 cm (a, b).

Taste papilla densities in *H. ocellatum* are highly variable between regions, with the maxillary oral valve (110 papillae cm^−2^) and anterior dorsal region of the oral cavity (103 papillae cm^−2^) having the highest concentrations compared with the mandibular oral valve (56 papillae cm^−2^) and anterior ventral region of the oral cavity (63 papillae cm^−2^) (Figure 20). Other regions of the oropharynx vary between 49 and 83 papillae cm^−2^ and 26 and 49 papillae cm^−2^ in the dorsal and ventral oropharynx, respectively (Table 3). The total number of taste papillae is 1,354.

### 3.6 Requiem sharks (Carcharhinidae)

#### 3.6.1 Morphology of taste papillae

**FIGURE 21:**
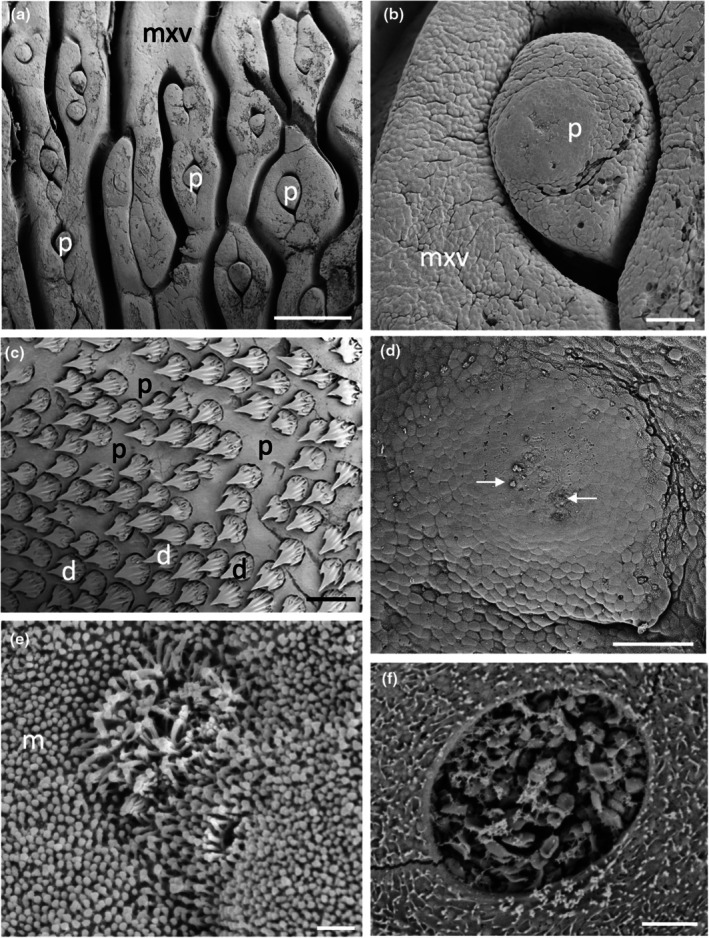
Taste papillae in the blacktip reef shark, *Carcharhinus melanopterus* viewed using scanning electron microscopy. (a) Taste papillae (p) within the folds of the maxillary oral valve (mxv). (b) Higher power of a taste papilla within the maxillary oral valve (mxv). (c) Type II papillae (p) amongst oral denticles (d) from the lateral side of the ventral oral cavity. (d) Higher power Type II taste papilla from the central dorsal oral cavity showing clumps of long microvilli (arrows) within the apical region. (e) High power of the long microvilli shown in (d). (f) Oval depression in the epithelial cell surface containing Type II‐like taste buds of teleosts. m, microvilli. See Figure 4 for other abbreviations. Scale bars: 0.5 mm (a); 50 μm (b); 0.5 mm (c); 50 μm (d); 1 μm (e); 2 μm (f).

**FIGURE 22:**
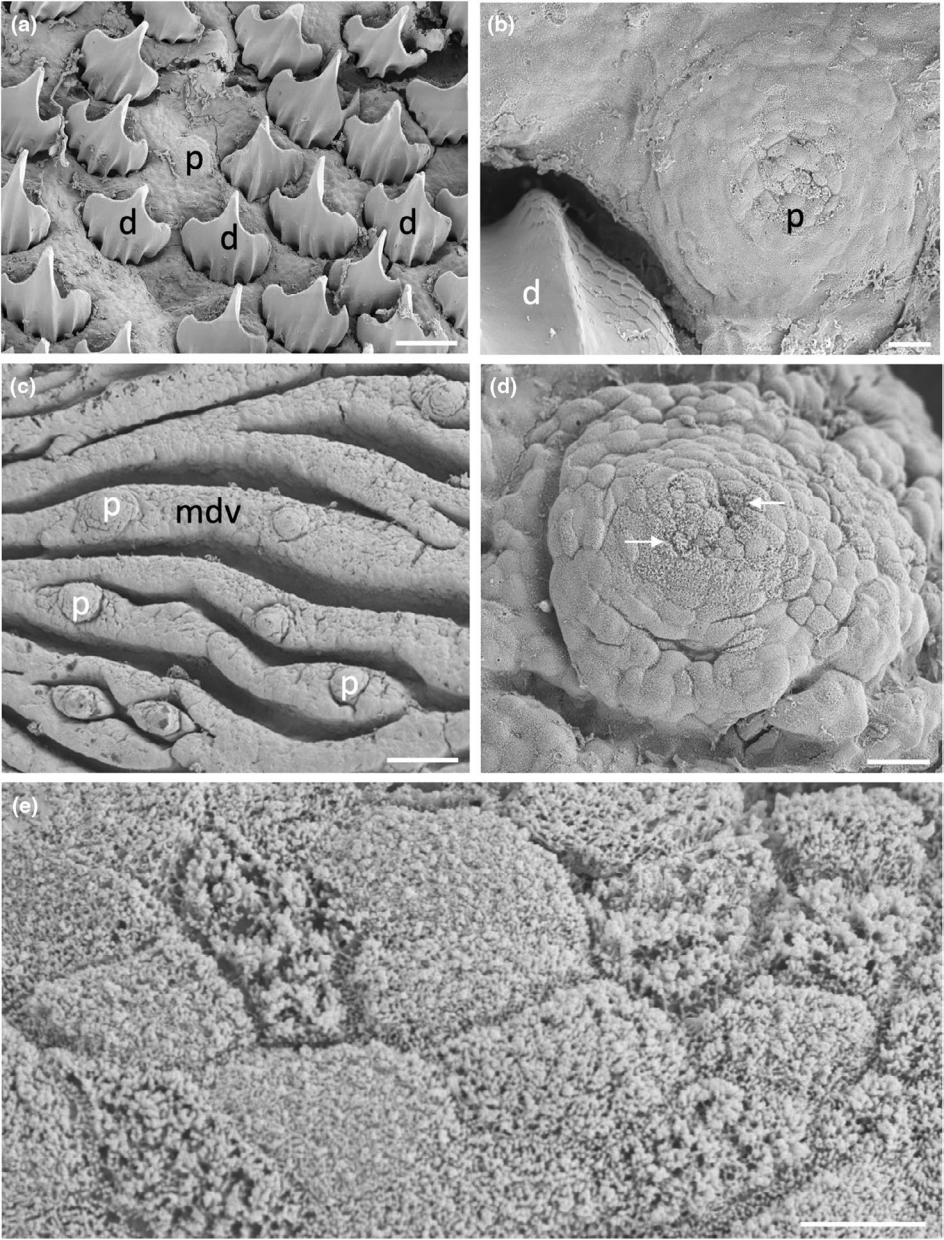
Taste papillae in the sicklefin shark, *Negaprion acutidens* viewed using scanning electron microscopy. (a) The central dorsal oral cavity showing a taste papilla (p) amongst oral denticles (d). (b) Higher power of a taste papilla in the central dorsal oral cavity showing a flattened apical region with numerous depressions surrounding selected epithelial cells. (c) Taste papilla within the mandibular valve (mdv). (d) High power of a single taste papilla within a mandibular oral valve (mdv). (e) Tufts of larger microvilli projecting from the surface of select taste cells on the apical tip of a taste papilla. Scale bars: 200 μm (a); 20 μm (b); 0.25 mm (c); 20 μm (d); 5 μm (e).

Two species of requiem sharks were sampled: the blacktip reef shark, *Carcharhinus melanopterus*, and the sicklefin shark, *Negaprion acutidens*. The oral valves of *C. melanopterus* are U‐shaped and taper at the edges. To the naked eye, the valves appear to have a smooth, flat surface with a straight border, although at the level of the SEM, they appear as a series of epidermal folds (Figures 21 and 22). Type I papillae surrounded by a basal depression are located on the elevated surfaces of the oral valves (Figure 21a,b). Microvilli protrude above the surface of the epithelium at the apical tip of the papilla. The sensory taste papillae, from which the microvilli originate, may be adjacent or separated by non‐sensory cells, resulting in areas of high microvilli density being dispersed over the apical surface. These papillae are comparable to Type I taste buds of teleost fishes (Reutter et al., 1974) since they are located on the oral valves and their base lies beneath the surface of neighbouring epithelial cells. They do not, however, protrude above the surrounding epithelial surface at an angle of 45°, but instead sit only slightly above its surface.

Less prominent papillae are located throughout the rest of the oropharyngeal region amongst a dense arrangement of oral denticles (Figure 21c) comparable to those of the external shark skin. These papillae are 203 ± 4 μm (*n* = 61) in diameter (Table 2). Microvilli are located on the apical tip either protruding (Figure 21d,e) or located in depressions covering ~0.7% of the papilla surface area. Microvilli are also seen in pits within the flat oropharyngeal epithelia (Figure 21f) located between oral denticles.

The oropharyngeal cavity of *N. acutidens* is very similar to that of *C. melanopterus*. The oral valves are U‐shaped tapering at the edges and are comprised of a series of epidermal folds (Figure 22c), which contain oral papillae. Each papilla is surrounded by a shallow basal depression (Figure 22b,c). At the apical surface of the papillae, microvilli protrude either adjacent to each other or are separated by non‐sensory cells (Figure 22e). Shallow papillae are found in the oropharyngeal cavity amongst a dense arrangement of oral denticles but lack the depression observed to surround the papillae within the oral valves (Figure 22a). In contrast, their bases are level with the surrounding epithelium. As in other species examined, microvilli protrude in various groups over their apical surface covering ~1.0% of the papilla area (Figure 22d,e, Table 2). No Type III taste buds were observed using SEM. Papillae are 152 ± 5 μm (*n* = 44) in diameter (Table 2).

#### 3.6.2 Habitat, diet, and distribution of taste papillae

The black‐tip reef shark, *C. melanopterus*, is often found in shallow water over reefs and sand flats and commonly in mangroves, while the sicklefin lemon shark, *N. acutidens*, is commonly found close to the bottom of the water column in shallow, sandy lagoons, and turbid mangrove swamps (Last & Stevens, 2009). Both species feed mainly on fishes, cephalopods, and crustaceans (Last & Stevens, 2009) and all have been observed to bite prey with their sharp triangular teeth, shake it, then bite it again repeatedly as the item enters the mouth and is consumed. Larger sharks would consume prey in fewer bites, so the mode of ingestion is likely dependent on prey size.

**FIGURE 23:**
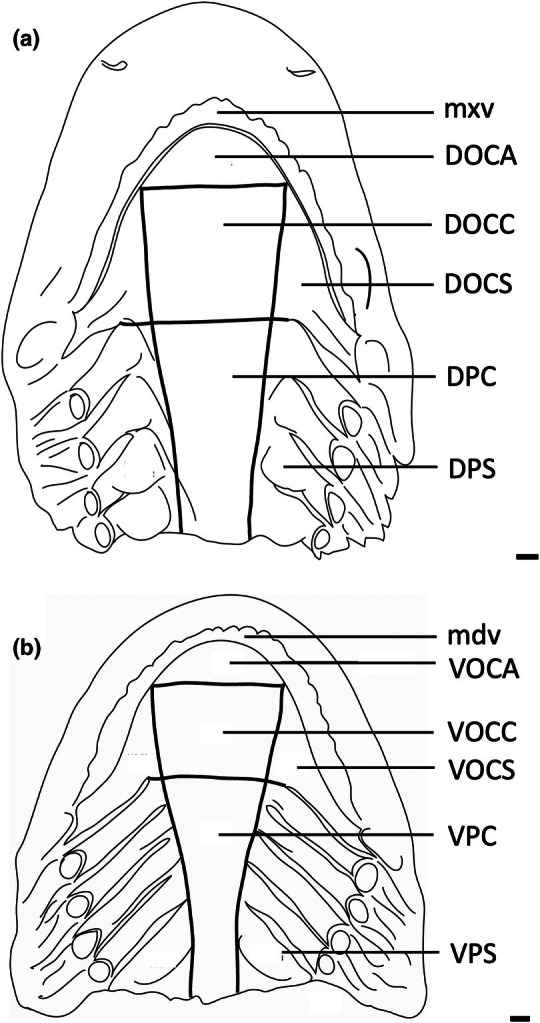
Mean oral papillae densities per cm^2^ ± SE for the various oropharyngeal regions of *Carcharhinus melanopterus* (*n* = 3, TL 933–1030 mm) in the maxilla (a) and mandible (b). Dots represent prominent taste papillae observed at the level of the light microscope. Maxillary oral valve (mxv). Mandibulary oral valve (mdv). See Figure 6 for other abbreviations. Scale bars: 1.0 cm (a, b).

**FIGURE 24:**
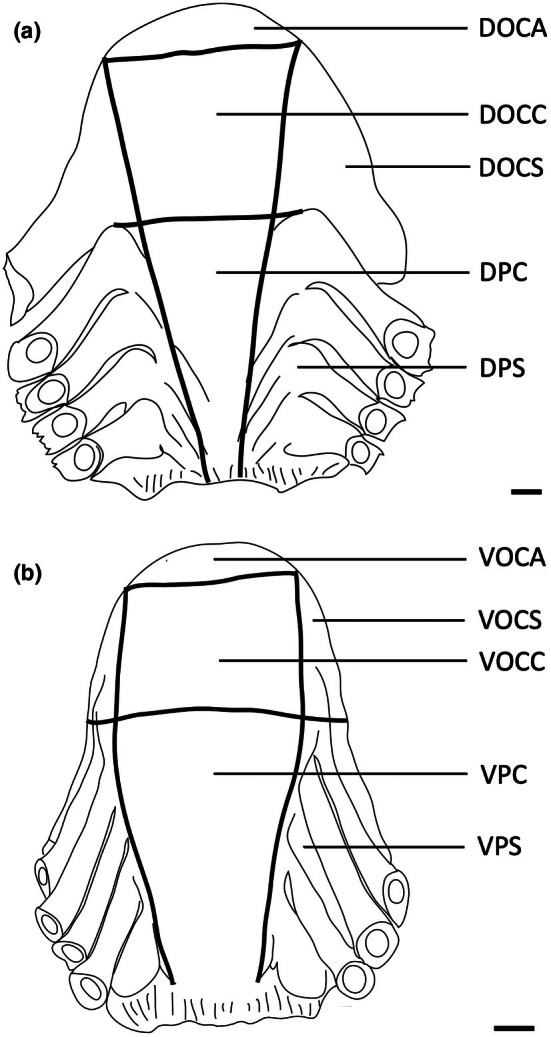
Mean oral papillae densities per cm^2^ ± SE for the various oropharyngeal regions of *Negaprion acutidens* (*n* = 1, TL 1209 mm) in the maxilla (a) and mandible (b). Dots represent prominent taste papillae observed at the level of the light microscope. Maxillary and mandibulary oral valves are not shown. See Figure 6 for other abbreviations. Scale bars: 1.0 cm (a, b).

Papillae are most dense on the oral valves with densities of 215 papillae cm^−2^ and 159 papillae cm^−2^ within the maxillary and mandibulary oral valves, respectively, in *C. melanopterus* and 83 papillae cm^−2^ and 90 papillae cm^−2^ within the maxillary and mandibulary oral valves, respectively, in *N. acutidens* (Figures 23 and 24, Table 3). Small papillae are distributed throughout the oropharyngeal epithelium, although there is such a dense arrangement of oral denticles, little space appears to be available for them to form. Oropharyngeal densities of papillae are slightly less in the central regions (*C. melanopterus*: 10–33 papillae cm^−2^; *N. acutidens*: 28–37 papillae cm^−2^) than towards the sides of the cavity (*C. melanopterus*: 29–58 papillae cm^−2^; *N. acutidens*: 37–75 papillae cm^−2^) and are slightly more concentrated in the anterior region (*C. melanopterus*: 38–58 papillae cm^−2^; *N. acutidens*: 40–116 papillae cm^−2^) (Table 3). Total papillae counts for *C. melanopterus* and *N. acutidens* are 9,235 ± 1464 and 11,890, respectively.

## 4 DISCUSSION

### 4.1 Arrangement of substructures within the oropharyngeal cavity

The oral cavity of all species examined possesses a mosaic‐like pavement of pentagonal and hexagonal stratified squamous epithelial cells of ~10 μm in diameter. The surface of these cells comprises dense patterns of microvilli and some microplicae, extensions of the membrane that increase surface area for metabolic exchange (Collin & Collin, 2006). Taste papillae of various types and sizes are embedded within or project from the epithelium and are dispersed over the oropharyngeal cavity and gill bars, often intermingled with oral denticles (Atkinson & Collin, 2012; Poscai et al., 2021, 2022; Rangel et al., 2017). In selachians, the taste papillae also extend over the maxillary and mandibulary oral valves in high concentrations, although they appear to be absent from the oral valves of many batoids (Gudger, 1931) with the exception of the two trygonorrhid species examined in this study. The oral (or breathing) valves are used to regulate respiratory currents to prevent regurgitation of water during expiration and are typically found in both cartilaginous and bony fishes that impale prey using their teeth and hold them before swallowing (Gudger, 1931; Whitear & Moate, 1994a). The batoid oral valves, particularly the maxillary valve, are very prominent flap‐like structures that extend some distance from the jaw line with a free edge. In selachians, however, oral valves are less prominent and often possess a free margin but would not be considered flap‐like as they are fixed to the underlying epithelium in the midline. It is proposed that the oral valves contribute less to the regulation of respiratory currents in these species, which use ram ventilation (Wegner & Graham, 2010), and are instead important as a region for increased gustatory perception. The high density of taste papillae over the oral valves in orectolobids, which are not ram ventilators but must still make a rapid decision to ingest prey following a strike, provides a relatively high level of gustatory perception, which may be initially ‘primed’ with the aid of water drawn also into the nares and over the olfactory receptors (Theiss et al., 2009).

### 4.2 Morphological characterization of taste buds

Following the descriptions of Reutter et al. (1974), the greater protrusion above the epithelium and location of the oral valve papillae within our study species, we consider these taste papillae to be Type I taste buds and those of the oral cavity and pharynx to be Type II taste buds. The higher density of Type I taste buds in the far anterior region and their absence in the posterior region of the oropharynx is also evident in numerous teleosts including cardinal fishes (Fishelson et al., 2004). The presence of Type III taste buds was not definitively confirmed. This was partly because of the appearance of the epithelial surface in the oropharynx, which was often folded or uneven when preserved and the fact that we did not histologically section large numbers of these taste papillae. Type III taste buds are differentiated by their location within a pore on an otherwise flat region of cornified, desquamating epithelium and an aggregation of microvilli (Reutter et al., 1974). However, comparable structures on the apical tips of oral papillae were identified in our study, where microvilli were observed in depressions in the epithelial surface, that is, in the flat epithelia of the oropharyngeal cavity of *C. melanopterus* and *G. australis*, but further studies are required to characterize these taste buds. Tufts of microvilli in pits have also been found within the flat, cornified, desquamating oral and pharyngeal epithelia of teleost fishes (Reutter et al., 1974) and noted in snake taste papillae, where microvilli protrude within a pit located at the apical tip of the taste bud (Nishida et al., 2000). The miniature Type IV taste buds reported in a number of cardinal fish species (*Apogon* sp.) reported by Fishelson et al. (2004) were not observed, although relatively small papillae were identified and quantified in the estuary stingray, *Hemitrygon fluviorum* and the blue‐spotted mask ray, *Neotrygon kuhlii* (Table 2), where five small papillae often surround large papillae (only observed using scanning electron microscopy).

Although the gross morphological characterization of taste buds established for teleosts (Reutter et al., 1974) reveals some potential homologies in cartilaginous species, other criteria are now required to differentiate the anatomical and functional diversity of taste buds in fishes. These could include ultrastructure (Lee et al., 2018), immunohistochemistry including ecto‐ATPase activity (Kirino et al., 2013; Yee et al., 2001), electrophysiological responses (taste preferences and sensitivity, Caprio, 1988; Kasumyan & Døving, 2003), innervation and connectivity, and molecular investigation of taste receptor genes (Shi & Zhang, 2006). These approaches will enable a more systematic study of the evolution of gustatory receptors.

### 4.3 The distribution of taste papillae and implications for food manipulation

The density of taste papillae within the oropharynx of the elasmobranchs examined is heterogeneous, with the oral valves and the anterior region typically possessing higher mean densities than the remainder of the oropharynx (especially in selachians) and the lateral regions or sides having higher densities than the central regions (Table 3). These findings suggest that based on the higher number of gustatory signals, elasmobranchs make decisions early on whether to ingest food based primarily on when the prey is held within their teeth or being crushed. The highest densities found within the oral valves and the anterior oral cavity (58–215 papillae cm^−2^) of all the selachians examined may also reflect the need to make a rapid decision on the palatability of their fast‐moving diets of bony fishes, small sharks, and stingrays (*Carcharhinus melanopterus* and *Negaprion acutidens*) and teleost fishes and octopi (*Orectolobus maculatus* and *O. ornatus*), although different feeding strategies are utilized (predatory strike versus sit‐and‐wait, respectively). The relatively lower densities of taste papillae within the remainder of the oropharyngeal region presumably permit a final taste assessment when the item is held in the mouth, until it is subsequently rejected or consumed. A similar distribution pattern is found in teleost fishes (Fishelson et al., 2004). Although not yet established for elasmobranchs, in catfishes the facial nerve innervates the taste buds of the body skin, lips, and anterior part of the mouth and is associated with the localization of food, which assists in triggering the pick‐up reflex, while the glossopharyngeal (nIX) and vagal (nX) nerves innervate taste buds of the posterior part of the mouth and gill arches and control the swallowing reflex (Atema, 1971).

While the elasmobranchs examined in this study all possess taste papillae, which concentrate in areas of food manipulation including the jaws and anterior regions of the oral cavity, taste papillae densities are low in comparison to teleost fishes. Maximum densities ranged from 73 to 175 papillae cm^−2^ in batoids to 33–215 papillae cm^−2^ in selachians irrespective of oropharyngeal region, while maximum papilla densities are appreciably higher in teleosts, i.e., 300–500 papillae cm^−2^ in the catfish, *Ictalurus natalis* (Atema, 1971), 2,000–4,000 papillae cm^−2^ in *Salvelinus* spp. (Hara et al., 1993), 17,000 papillae cm^−2^ in the tench, *Tinca tinca* (Fishelson et al., 2004) with densities reaching as high as 30,000 papillae cm^−2^ in some cyprinids (Gomahr et al., 1992). In broad terms, higher densities of taste papillae are found in benthic and benthopelagic teleost species compared with open water species, which may reflect the importance of gustation in species frequenting low light environments (Gomahr et al., 1992; Hara et al., 1994; Komada, 1993). Unfortunately, this correlation was not found in the elasmobranchs sampled in this study, where there was no significant relationship between maximum papillae density and depth (Tables 1 and 3), although future comparisons with much deeper water species (i.e. members of the *Etmopterus, Centroscymnus, Somniosus, Scymnodon* genera) may still support such a correlation. In fact, maximum density (which primarily was found over the oral valves and anterior regions but was also found on the sides of the pharynx in some species) was not correlated with either phylogenetic group (batoids versus selachians) or habitat (benthic, benthopelagic or pelagic). Interestingly, the two species of orectolobids (*Orectolobus maculatus* and *O. ornatus*) possess the lowest densities of papillae, which may reflect their nocturnal sit‐and‐wait ambush strategy, where their reliance on other sensory modalities such as electroreception (Egeberg et al., 2014; Theiss et al., 2011), the lateral line (Theiss et al., 2012) and olfaction (Theiss et al., 2009) may be more important for prey capture, where prey are ingested whole and by suction in the relatively low light conditions in which they live (up to 250 m).

The regions of high papilla density may reflect increased gustatory resolution, which must play an important role in each species' decision to ingest or reject prey. The spacing of the taste papillae may also be determined by the size of the preferred prey items, the density and packing of teeth and oral denticles (Atkinson & Collin, 2012; Poscai et al., 2022; Rangel et al., 2017) and the mode of feeding. Further work is required to assess the distribution of taste papillae, especially the regions of high gustatory ‘acuity’ with respect to preferred diet, feeding strategy, and food manipulation within the oropharynx.

### 4.4 Total number of taste papillae and gustatory sensitivity

The total number of taste papillae in our 11 species of elasmobranchs varied from 1,354 papillae in the benthic epaulette shark, *Hemiscyllium ocellatum* (occupying 0.018% or 521,148 μm^2^ of the gustatory area) to 20,317 papillae in the benthopelagic *Hemitrygon fluviorum* (occupying 0.05% or 3,855,035 μm^2^ of the gustatory area). Within this range, there was a significant difference between the five benthic batoid species, with a range of 2,119 to 9,834 papillae, and the benthopelagic *H. fluviorum* with 20,317 papillae. A similar increase in the total number of papillae was evident between the three benthic selachians, with a range of 1,354 to 1,993 papillae, and the pelagic selachians, with a range of 9,235 to 11,890 papillae. Therefore, in the small sample size of elasmobranchs presented here, higher numbers of papillae occur in pelagic/benthopelagic species than purely benthic species, a relationship that was strongest within the selachians. A similar range in the total number of taste buds occurs in teleost fishes, that is, 6,600 taste buds in the minnow, *Pseudorasbora parva* (Kiyohara et al., 1980), 7,500 taste buds in gobiid fishes (Fishelson & Delarea, 2004), 15,300 taste buds in the Amago salmon, *Oncorhynchus rhodurus* (Komada, 1993) and up to 20,000 taste buds within the oral cavity in the catfish *Ictalurus notatus* (Atema, 1971). Interestingly, this entire range (1,660–24,600 taste buds) is found across 10 species of closely related cardinal fishes of similar size (Fishelson et al., 2004).

Although the total number of taste papillae in these pelagic/benthopelagic species may indicate some form of gustatory specialization, the dimensions of the oropharynx, the diameter (and potentially type) of the taste papillae, and the proportion of the oropharynx they occupy should also be taken into account. Similarly, the area occupied by the apical microvilli of all oropharyngeal taste papillae, which ultimately transduce a gustatory stimulus that is conveyed to the brain, needs to be included in comparative discussions about which species may be considered gustatory specialists. Interestingly, all of the species with high numbers of papillae are the largest in size (total length, TL): *A. rostrata* (8,630 papillae—750 mm TL), *H. fluviorum* (20,317 papillae—918 mm TL), *N. kuhlii* (9,834 papillae—450 mm TL, estimated using Fishbase), *C. melanopterus* (9,235 papillae—1,030 mm TL) and *N. acutidens* (11,890 papillae—1,380 mm TL). Predictably, these same species also possess the highest areal measures of gustatory tissue, with a range of 2,092 × 10^3^ to 3,855 × 10^3^ μm^2^, compared with all other species, which range between 331 × 10^3^ and 775 × 10^3^ μm^2^ (Table 2). In comparison to the teleosts analysed, gobies have sensory areas in the region of 80 × 10^3^ μm^2^, and two species of cardinal fishes of 75 mm and 85 mm total length possess sensory areas of 50 × 10^3^ and 300 × 10^3^ μm^2^, respectively (Fishelson et al., 2004). Elasmobranchs, therefore, have very large sensory areas dedicated to gustatory input when compared with teleosts. However, these high papilla numbers may not necessarily indicate high gustatory acuity (Glaser, 1966), but rather indicate a level of gustatory sensitivity. Sensitivity is expected to reduce over the lifetime of each species, given that elasmobranchs maintain the same number of papillae (with a concomitant decrease in density) as they grow (Atkinson et al., 2016). Future investigation of the innervation (number of gustatory nerve axons) in regions of high density and throughout the oropharynx will uncover the relationship between gustatory resolution and sensitivity, respectively, and also more accurately assess the number of inputs from the solitary chemosensory cells.

## 5 CONCLUSIONS

The oropharynx of six species of batoids and five species of selachians is found to have a heterogeneous distribution of at least two types of taste papillae (in addition to solitary chemosensory cells) with the highest densities typically found just posterior to the teeth and tooth plates (including the oral valves) in areas used for food mastication in most species. Total numbers of taste papillae also vary with a benthopelagic batoid and two pelagic selachians having the highest populations. The terms gustatory resolution and sensitivity are introduced to understand the relationships between the need for localized discrimination of prey, oral food manipulation and palatability, and areal coverage of taste papillae and their apical microvilli, respectively. Studies in cartilaginous fishes provide a unique opportunity to trace the evolution of gustation.

## AUTHOR CONTRIBUTIONS

Both CJA and SPC conceived of the project and its design and methodology. CJA did much of the data acquisition and analyses, and both authors were involved in interpretation. CJA drafted initial versions of the manuscript, and SPC critically revised it for publication.

## Data Availability

The data that support the findings of this study are available from the corresponding author upon reasonable request.
